# A Consensus Genetic Map for *Pinus taeda* and *Pinus elliottii* and Extent of Linkage Disequilibrium in Two Genotype-Phenotype Discovery Populations of *Pinus taeda*

**DOI:** 10.1534/g3.115.019588

**Published:** 2015-06-11

**Authors:** Jared W. Westbrook, Vikram E. Chhatre, Le-Shin Wu, Srikar Chamala, Leandro Gomide Neves, Patricio Muñoz, Pedro J. Martínez-García, David B. Neale, Matias Kirst, Keithanne Mockaitis, C. Dana Nelson, Gary F. Peter, John M. Davis, Craig S. Echt

**Affiliations:** * School of Forest Resources and Conservation, University of Florida, Gainesville, Florida, 32611-0410; † Southern Research Station, USDA Forest Service, Saucier, Mississippi, 39574; ‡ National Center for Genome Analysis Support, Indiana University, Bloomington, Indiana 47408; § Department of Biology, University of Florida, Gainesville, Florida 32611; ** Department of Pathology, Immunology and Laboratory Medicine, University of Florida, Gainesville, Florida 32603; †† Genetics Institute, University of Florida, Gainesville, Florida 32608; ‡‡ RAPiD Genomics LLC, Gainesville, Florida 32601; §§ Department of Agronomy, University of Florida, Gainesville, Florida 32608; *** Department of Plant Sciences, University of California, Davis, Davis, California, 95616; ††† Pervasive Technology Institute, Indiana University, Bloomington, Indiana 47408

**Keywords:** pine, linkage mapping, linkage disequilibrium, population structure

## Abstract

A consensus genetic map for *Pinus taeda* (loblolly pine) and *Pinus elliottii* (slash pine) was constructed by merging three previously published *P. taeda* maps with a map from a pseudo-backcross between *P. elliottii and P. taeda*. The consensus map positioned 3856 markers via genotyping of 1251 individuals from four pedigrees. It is the densest linkage map for a conifer to date. Average marker spacing was 0.6 cM and total map length was 2305 cM. Functional predictions of mapped genes were improved by aligning expressed sequence tags used for marker discovery to full-length *P. taeda* transcripts. Alignments to the *P. taeda* genome mapped 3305 scaffold sequences onto 12 linkage groups. The consensus genetic map was used to compare the genome-wide linkage disequilibrium in a population of distantly related *P. taeda* individuals (ADEPT2) used for association genetic studies and a multiple-family pedigree used for genomic selection (CCLONES). The prevalence and extent of LD was greater in CCLONES as compared to ADEPT2; however, extended LD with LGs or between LGs was rare in both populations. The average squared correlations, r^2^, between SNP alleles less than 1 cM apart were less than 0.05 in both populations and r^2^ did not decay substantially with genetic distance. The consensus map and analysis of linkage disequilibrium establish a foundation for comparative association mapping and genomic selection in *P. taeda* and *P. elliottii*.

More than one billion *Pinus taeda* L. (loblolly pine) seedlings are planted each year in the United States in 13 million hectares of plantations that extend from Eastern Texas to Delaware (McKeand *et al.* 2003; Smith *et al.* 2007). Southern pine plantations, composed primarily of *P. taeda* and *P. elliottii* (slash pine), supply 60% of the wood products in the United States and 18% worldwide (Prestemon and Abt 2002).

A high-density consensus linkage map that is primarily based on polymorphisms within genes will be useful for all areas of genomic research in these economically important *Pinus* species. Genomic selection, which aims to predict breeding values from the summed effects of genome-wide genetic markers (Meuwissen *et al.* 2001), has the potential to accelerate the current breeding cycle of *P. taeda* from 12 to 20 years to less than 7 years (Resende *et al.* 2012a). A high-density genetic map may be used to design low-density panels of markers that reduce genotyping costs for genomic selection without sacrificing prediction accuracy (Habier *et al.* 2009). A consensus genetic map may also be used to compare the locations of marker-trait associations in independent populations. For example, Westbrook *et al.* (2015) used a consensus map to compare the locations of QTL associated with resin canal number in a pseudo-backcross between loblolly pine and slash pine to the locations of single nucleotide polymorphisms (SNPs) associated with the same trait in a complex pedigree of loblolly pine.

Quantifying the genome-wide extent of linkage disequilibrium (LD) within genotype-phenotype discovery is relevant for association genetics and genomic selection. LD or the nonindependence of segregating alleles at different genomic loci may arise from two loci being in close proximity on a chromosome, thus reducing the probability of recombination between them (Flint-Garcia *et al.* 2003).

Statistical power to detect an association between a marker and a trait is inversely proportional to the squared correlations (r^2^) between alleles at a marker locus and the causal variant (Pritchard and Przeworski 2001). Thus, quantifying the extent of LD is useful for knowing marker densities required to represent nonrecombining haplotype segments within discovery populations (Yan *et al.* 2009). LD among distant loci on the same chromosome or loci on different chromosomes may also occur because of subpopulation structure, kinship, inbreeding, directional selection, and epistasis (Gaut and Long 2003). Estimating LD between distant loci on the same chromosome or on different chromosomes is useful for detecting the possibility of false-positive associations (Platt *et al.* 2010). Within outcrossing populations of conifers with large effective population sizes, r^2^ decays rapidly to less than 0.1 over 500 to 1500 bases (Brown *et al.* 2004; Neale and Savolainen 2004; Pavy *et al.* 2012a). However, within multi-family pedigrees used for genetic association and genomic selection studies, r^2^ is expected to decay over greater distances proportional to levels of relatedness (Flint-Garcia *et al.* 2003).

In the present study, a consensus genetic map was constructed by merging three gene-based linkage maps for *P. taeda* (Echt *et al.* 2011; Martínez-García *et al.* 2013; Neves *et al.* 2014) with a linkage map from a pseudo-backcross between *P. elliottii* and *P. taeda* (Westbrook *et al.* 2015). The consensus map positioned 3856 markers via genotyping 1251 individuals from three full-sib populations and one haploid population. Improved functional annotations of mapped genes were obtained by aligning the partial length expressed sequence tags (ESTs) used for marker discovery against longer *P. taeda* transcript assemblies from RNA-seq data (NCBI BioProject PRJNA174450) (Wegrzyn *et al.* 2014). The consensus map was used to compare the genome-wide extent of LD in ADEPT2, a population composed of unrelated individuals that has been used for association genetic studies (Quesada *et al.* 2010; Eckert *et al.* 2010; Cumbie *et al.* 2011), and CCLONES, a complex multi-family population that has been used for genomic selection studies (Resende *et al.*, 2012a,b; Westbrook *et al.* 2013, 2015).

## Materials and Methods

### Linkage maps used to construct the consensus map

A consensus map was constructed by merging two composite maps from the *P. taeda* QTL and BASE pedigrees (Echt *et al.* 2011; Martínez-García *et al.* 2013) with a BC1 map from a (*P. taeda* × *P. elliottii*) × *P. elliottii* pseudo-backcross and a haploid map from the *P. taeda* clone 10-5 (Neves *et al.* 2014). The composition of marker types in the four input maps is given in Table 1. The first QTL-BASE map (QTL-BASE1) contained 460 markers (Supporting Information, File S1) genotyped in single F2 cohorts of the QTL and BASE pedigrees (Echt *et al.* 2011). The second QTL-BASE map (QTL-BASE2) contained 2466 markers (File S2) genotyped in two F2 full-sib cohorts in each of the BASE and QTL pedigrees (Martínez-García *et al.* 2013). Both QTL-BASE maps were constructed in JoinMap with regression mapping (Van Ooijen 2011). Linkage group (LG) numbers and orientation in all input maps were modified to match the historical designations of Echt *et al.* (2011).

**Table 1:** Composition of input maps used to construct consensus genetic map for *Pinus taeda* and *Pinus elliottii*

Input Map	Cohort Structure	*N*_ind_	Marker Type	*N*_markers_ Original	*N*_markers_ Selected	Avg. GIC	Weight	Length, cM
QTL-BASE1	BASE	97	SSR	233	197	402	0.21	1413
	QTL	170	RFLP	123	113			
	**Total**	**267**	ESTP	104	96			
			**Total**	**460**	**406**			
QTL-BASE2	BASE1	92	SNP	2307	1895	462	0.24	1476
	BASE2	110	RFLP	124	124			
	QTL1	180	ESTP	35	35			
	QTL2	307	**Total**	**2466**	**2054**			
	**Total**	**689**						
BC1	1 cohort	490	SNP	803	803	941	0.49	1378
10-5	1 cohort	72	SNP	2776	1359	121	0.06	1910
			PAV	65	16			
			**Total**	**2841**	**1375**			

Data source for input map: QTL-BASE1 (Echt *et al.* 2011), QTL-BASE2 (Martínez-García *et al.* 2013), BC1 (Westbrook *et al.* 2015), 10-5 (Neves *et al.* 2014); *N*_ind_, number of individuals in the mapping populations; Marker type: SNP, single nucleotide polymorphism; PAV, presence/absence variants of genes; RFLP, restriction fragment length polymorphisms, ESTP, expressed sequence tag polymorphism; SSR, simple sequence repeat; *N*_markers_ original, total number of markers in the original input map, including all redundancies, by marker type and total; *N*_markers_ selected, number of nonredundant markers selected for maximum informativeness in consensus mapping, by marker type and total; Avg. GIC, input map’s average marker genotype information content; Weight, input map weight value used to resolve marker order conflicts in consensus mapping, scaled in proportion to Avg. GIC; Length, input map’s genome length in cM_(Kosambi)_ units.

### Reconstruction of the BC1 input linkage map

A composite map of the BC1 and 10-5 populations was presented in Westbrook *et al.* (2015). Due to large differences in progeny sizes between these populations (490 diploid individuals in BC1 *vs.* 72 haploid megagametophytes in 10-5), we reconstructed a genetic map of the BC1 population separately from the 10-5 map prior to its integration into the current consensus map. The BC1 pseudo-backcross population originated from controlled pollination of a *P. taeda* × *P. elliottii* var. *elliottii* F1 hybrid with pollen from a second *P. elliotti* var. *elliottii* individual (Muñoz *et al.* 2011). Full-sib BC1 progeny, their parents, and the maternal *P. elliottii* grandmother were genotyped at 4861 SNPs discovered within ESTs with an Illumina Infinium assay designed for *P. taeda* (Eckert *et al.* 2010). Loci that were monoallelic, missing parental genotypes, displayed significant segregation distortion at *P* < 0.001, or had more than 5% genotyping error rate as inferred from parental genotypes were discarded. For loci that contained genotype errors in less than 5% of the individuals, the erroneous genotypes were recoded as missing data. For ESTs containing more than one SNP, the marker with the highest genotype information content (described below) was selected for mapping. The BC1 map containing 803 SNPs (File S3) was constructed in JoinMap v. 4.1 (Van Ooijen 2011) by specifying the cross-pollinated (CP) population type, a linkage group LOD score threshold of 6, and the Kosambi mapping function. Map positions from the third round of regression mapping were used.

### Reconstruction of the 10-5 linkage map

The 10-5 map was originally constructed from exome resequencing of 72 haploid megagametophytes from a single tree and contained 2841 markers (Neves *et al.* 2014). Large regions of reversed marker order were observed on 5 of 12 linkage groups, when the 10-5 map was compared to a previously published genetic map of the *P. taeda* QTL population (Eckert *et al.* 2010). Reversals on these five LGs (4, 5, 6, 7, and 8) were also observed when the marker order of the 10-5 map was compared with the BC1 map (Figure S1) and the QTL-BASE2 map (Figure S2). By contrast, strong colinearity was observed among QTL-BASE1, QTL-BASE2, and BC1 maps (Figure S3 and Figure S4). Considering that the 10-5 map was constructed from genotyping a relatively small population (Table 1) at a low median sequence depth of 2.7× (Neves *et al.* 2014), marker order reversals in the 10-5 map were likely attributable to genotyping or mapping errors instead of cytological rearrangements (Maleipaard *et al.* 1997). Based on this hypothesis, the 10-5 map was reconstructed in three steps that took advantage of the “fixed order” and “start order” map building functions in JoinMap v.4.1: (1) define a fixed order for a subset of reference loci obtained from two of the other mapping pedigrees; (2) define a robust start order for a subset of loci on a preliminary 10-5 map built from markers having the lowest levels of missing genotypes and suspect linkages; and (3) specify limited sets of fixed order and start order loci to aid reconstruction of the 10-5 map.

First, a reference marker order was obtained by merging the BC1 and QTL-BASE2 maps with MergeMap (Wu *et al.* 2011) to obtain a composite map that shared a greater number of markers with the 10-5 map as compared to the individual BC1 or QTL-BASE2 maps. For each LG, 6 to 19 loci were identified for use as fixed order reference loci based on their being separated by than more than 10 cM and having the highest genotype information content (described below) among neighboring loci.

Second, a preliminary 10-5 map was constructed from loci that had less than 12.5% missing genotypes to help define a starting marker order. Two rounds, not three, of regression mapping in JoinMap v. 4.1 were used to ensure that no loci had negative recombination intervals and that all loci had goodness-of-fit chi-square values greater than 3. The following mapping parameters were specified: a haploid population type; a linkage group LOD threshold = 6; mapping LOD threshold = 1.5; recombination threshold = 0.44; jump threshold = 3.0; ripple value = 1; and the Kosambi mapping function. After an initial round of mapping, loci were excluded from subsequent maps if they had a high frequency of suspect pairwise linkages, recombination fractions >0.60 and mapping LOD >1.5, high nearest neighbor fit values and low locus mean genotype probabilities, or if they had prominent order conflicts with the BC1/QTL-BASE2 reference map. Iterative exclusion of loci and remapping continued until there was no further improvement in alignment to the BC1/QTL-BASE2 reference map. Start order loci were then identified based on their being separated by more than 5 cM, having the highest genotype information content among neighboring loci, and having marker orders not in conflict with the BC1/QTL-BASE2 reference map order. Between 11 and 28 start order loci were selected for each LG.

Third, starting with genotype data for all loci reported by Neves *et al.* (2014), the final 10-5 map was reconstructed by specifying the fixed orders and start orders obtained from the first two steps. Iterative exclusion of loci and remapping were then performed using the parameters and protocols described in the second step above. Loci at the ends of LGs that were more than 10 cM apart from adjacent loci were also excluded. Mapping iterations continued until there was no further improvement in map quality as assessed by chi-square values for each LG or alignment to the BC1/QTL-BASE2 reference map. Table S1 summarizes the number of start order loci, fixed order loci, and markers used to reconstruct the 10-5 map (File S4).

### Marker selection for the consensus map

The majority of loci shared among the BC1, BASE-QTL2, and 10-5 maps were SNPs discovered within expressed sequence tags (ESTs). To construct a consensus map, SNP loci were merged based on EST names after omitting the nucleotide position of the SNP within the name. Nucleotide positions in the 10-5 map did not correspond to the positions in the QTL-BASE2 and BC1 maps because the SNPs were discovered in different populations and were based on different alignments. To merge the BC1 and 10-5 maps, which contained one SNP per EST, to the QTL-BASE2 consensus map, which contained one to three SNPs per EST, it was necessary to select the most informative SNP within ESTs. The QTL-BASE1 map was merged with the other maps via simple sequence repeats (SSR) and restriction fragment length polymorphisms shared with the QTL-BASE2 map.

Marker informativeness was measured with genotype information content (GIC) calculated as the effective number of genotypic classes (*G*_e_) times the number of individuals genotyped (*N*_indiv_). The effective number of genotypic classes for each marker was calculated as *G*_e_ = $1/\sum p_{j}^{2}$ where *p*_j_^2^ is the squared proportion of the *j*^th^ genotypic class. For biparentally heterozygous (hk × hk) loci, which segregate 1hh:2hk:1kk, *G*_e_ was calculated using only the homozygous (hh and kk) genotypic classes, and GIC was calculated using the total number of genotyped progeny. The heterozygous (hk) genotype was excluded from the calculation of *G*_e_ to maintain an inverse linear proportionality of GIC with segregation distortion chi-square values. For genes genotyped at more than one SNP marker, only the marker with the maximum GIC summed across population cohorts was retained in the QTL-BASE2 map. File S1, File S2, File S3, and File S4 contain the input maps with GIC used for marker selection.

### Map weights and construction of the consensus map

Constructing a consensus map directly from recombination frequencies in JoinMap is computationally time-consuming and infeasible for multiple populations and cohorts (Wenzl *et al.* 2006). Instead, the consensus map was constructed directly from the marker names and genetic distances in the input maps using two linear programming algorithms, MergeMap (Wu *et al.* 2011; http://www.mergemap.org) and LPmerge (Endelman and Plomion 2014; http://cran.r-project.org/web/packages/LPmerge/). MergeMap uses directed acyclic graphs of shared markers to merge input maps and resolves marker order conflicts by deleting the minimum number of marker occurrences from the input maps. LPmerge groups markers into positional bins and seeks the bin order that minimizes the root mean squared error between the input maps and consensus map. For both approaches, the resolution of marker order conflicts was informed by weighting input maps in proportion to the average GIC of the markers contained in each map. This weighting method assigned confidence to maps in proportion to the average number of individuals and genotypic classes genotyped and in inverse proportion to the average segregation distortion of the markers. For the QTL-BASE1 and QTL-BASE2 maps, which were constructed from two and four cohorts of the BASE and QTL populations, respectively, marker GIC was summed across all cohorts prior to averaging across markers. The average GIC for the *l*^th^ input map constructed from *k* cohorts and *j* markers was calculated as follows:

$${\bar{GIC}}_{jkl}=\frac{\sum_{k=1}^{k}GIC_{j\left(k\right)}}{N_{j\left(l\right)}}$$

where *GIC_j_*_(_*_k_*_)_ is the GIC of the *j*^th^ marker within the *k*^th^ cohort and *N_j(l)_* is the number of markers within the *l*^th^ map. Map weights were scaled from 0 to 1 by dividing ${\bar{GIC}}_{jkl}$ by the sum of the average GIC across maps, $\sum_{l=1}^{l}{\bar{GIC}}_{jkl}$. The centimorgan positions of markers varied between runs of MergeMap but did not vary between runs of LPmerge. To construct a consensus map in MergeMap, the average position of and standard error of marker positions were obtained from 100 replicate runs. Root mean squared errors (RMSE) in marker order between the consensus maps and the input maps and between the MergeMap and LPmerge consensus maps were calculated with the R package hydroGOF (http://cran.r-project.org/web/packages/hydroGOF/). The consensus map with the lowest average RMSE with the input maps was used for further analysis.

### Alignment of mapped genes to the *P. taeda* genome and transcriptome

Expressed sequence tags containing mapped markers (File S5) were aligned to *P. taeda* genome assembly version 1.01 (Neale *et al.* 2014) using GMAP (Wu and Watanabe 2005). For ESTs that aligned with more than one genomic scaffold or scaffolds that aligned with two or more ESTs on different LGs, the most precise alignment was chosen based on alignment length and DNA sequence identity. BlastN (Altschul *et al.* 1990) 2.2.27+ was used to align ESTs to transcript sequences assembled from RNA-seq reads of the *P. taeda* reference genotype 20-1010 and other genotypes (K. Mockaitis, data available in NCBI BioProject PRJNA174450). Predicted functions of coding sequences were provided from results of Delta-BlastP (Boratyn *et al.* 2012) alignments of complete transcript protein sequences to the *Arabidopsis thaliana* TAIR10 annotation protein set (Lamesch *et al.* 2011) and to the NCBI Conserved Domain Database (Marchler-Bauer *et al.* 2013).

### Spatial density of mapped genes along chromosomes

The density of markers along LGs was estimated with kernel density estimation in R (R Core Team 2012). Fixed bandwidths (*bw*) for the Gaussian kernel density estimator were calculated for each LG following the work of Silverman (1986):

*bw* = 0.9s/1.34*n*^−1/5^, where s is the SD of marker positions in cM and *n* is the number of markers per linkage group. Kernel density estimates were multiplied by *n* × *bw* to obtain the number of markers per bandwidth and by *n* to obtain the number of markers per centimorgan. To test for regions where marker density significantly deviated from random expectation, the observed numbers of markers per bandwidth were compared to the 95% C.I. of a Poisson distribution with mean and variance equal to *n*/(LG length/*bw*).

### Analysis of linkage disequilibrium in two populations of *P. taeda*

The consensus map was used to compare LD in CCLONES (Comparing Clonal Lines ON Experimental Sites), a pedigreed population of *P. taeda* used for genomic selection studies, and ADEPT2 (Allele Discovery of Economic Pine Traits 2), a population of unrelated *P. taeda* individuals used for association genetic studies. The CCLONES population was composed of 923 progeny in 68 full-sib families generated from circular mating among 54 first- and second-generation selections from breeding programs in Florida, the Atlantic coastal plain, and the lower Gulf states (Baltunis *et al.* 2007). The ADEPT2 population consisted of 427 distantly related *P. taeda* individuals sampled from throughout the species range (Eckert *et al.* 2010). Both populations were genotyped with an Illumina Infinium assay of 7216 SNPs discovered within ESTs (Eckert *et al.* 2010). Polymorphisms were detected at 3938 SNP loci within 3347 ESTs in ADEPT2 and at 4854 SNP loci within 4027 ESTs in CCLONES (Westbrook *et al.* 2013). Within ADEPT2 and CCLONES, respectively, 575 and 805 ESTs were genotyped at two or more SNP loci, enabling estimation of LD within genes.

LD between SNPs at different positions on the consensus map was estimated in both populations before and after adjusting r^2^ values for kinship or subpopulation structure with the R package LDcorSV (Mangin *et al.* 2012). Kinship was estimated from identity-by-descent proportions (IBD) expected from pedigree relationships (Henderson 1976). In CCLONES, IBD varied from 0 to 0.5; however, average IBD between all individuals was small (0.043) due to the fact that 80% of individuals were unrelated (Table S2). In ADEPT2, IBD was zero among distantly related individuals. Subpopulation structure was inferred from SNPs with minor allele frequencies greater than 0.05 with the program fastSTRUCTURE (Raj *et al.* 2014). In CCLONES, the number of subpopulation clusters (K) tested varied from 2 to 10 using 3037 SNPs. A continuous increase in marginal likelihood with increasing K was observed, indicating that subpopulation structure was weak or nonexistent (Figure S5). In ADEPT2, K = 2…7 were tested with 2910 SNPs, and K = 2 had the greatest marginal likelihood (Figure S5). The K = 2 structure matrix was not invertible; therefore, the structure matrix with three subpopulations (File S6), the second most likely K, was utilized to account for structure in the estimation of LD in ADEPT2. Kernal regression of r^2^ *vs.* genetic distance was performed with the ksmooth function in R. Plots of r^2^ within genes, within LGs, and between LGs were prepared in ggplot2 for R (Wickham 2009).

## Results

### Comparisons of the input maps used to construct the consensus genetic map for *Pinus taeda* and *Pinus elliottii*

Based on the hypothesis that large regions of reversed marker order on five LGs of the 10-5 map were artifacts of small population size and genotyping errors (see *Materials and Methods*), the 10-5 map was reconstructed by specifying limited sets of selected fixed order and start order loci, and then subsequently excluding poor-fitting loci through an iterative mapping procedure (Table S1). In total, 1466 markers from the original 10-5 map were excluded to reconstruct a 10-5 map containing 1375 markers (File S4). The reconstructed 10-5 map was collinear with the QTL-BASE2 and BC1 maps (Figure S6 and Figure S7).

The four input maps were merged with 69 to 497 markers that were shared between pairs of maps (Table S3). The QTL-BASE1 map, composed of simple sequence repeat (SSR), restriction fragment length polymorphism (RFLP), and expressed sequence tag polymorphism (ESTP) markers, did not share markers with the 10-5 and BC1 maps, which were composed primarily of SNPs (Table 1). The QTL-BASE1 map was integrated into the consensus map via markers shared with the QTL-BASE2 map. The 54 markers on LGs 7 and 12 from the QTL-BASE1 (Echt *et al.* 2011) (File S1) were excluded from the merge to create the consensus map because QTL-BASE1 shared only two markers with QTL-BASE2 on these LGs. A total of 412 SNP markers were excluded from the QTL-BASE2 map prior to consensus merging because they occurred within expressed sequences containing SNPs at other nucleotide positions that had higher genotype information content (Table 1).

### Comparisons of the consensus maps from two map merging algorithms

The consensus maps generated by MergeMap (File S7) and LPmerge (File S8) each contained 3856 markers and were strongly collinear with each other (Figure S8). The total length of the MergeMap consensus map (2305 cM) was 1.2- to 1.7-times longer than the lengths of the individual input maps, whereas the length of the LPmerge consensus (1802 cM) was within the range of the lengths of the input maps (1378–1910 cM) (Table 1). Where there was uncertainty in marker order between the consensus maps, LPmerge binned markers into the same map positions, whereas MergeMap assigned unique positions to most markers (Table S4). This nonbinning attribute of MergeMap accounts for the length expansion of its consensus map compared to the LPmerge map. The MergeMap consensus had lower average RMSE in marker order with the input maps for 10 of 12 LGs (LGs 1–10) as compared to the LPmerge consensus (Table S4 and Table S5). Therefore, the MergeMap consensus map was used for subsequent analyses.

### Summary of the consensus map and alignment to the *P. taeda* genome and transcriptome

**Figure 1:**
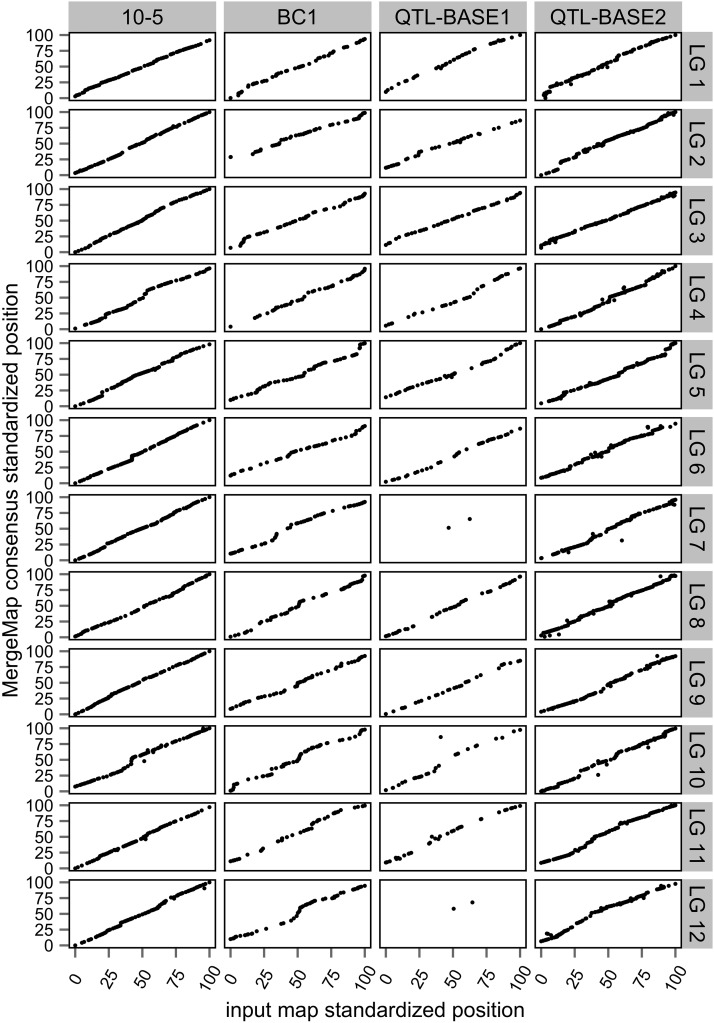
Comparisons of orders of shared markers between input maps and the MergeMap consensus genetic map of *Pinus taeda* and *Pinus elliottii*. Linkage group (LG) lengths were standardized to 100 units for comparison among maps.

Strong colinearity was observed between the MergeMap consensus and the four input maps (Figure 1). The consensus map positioned 3353 SNPs discovered within ESTs, 175 restriction fragment length polymorphisms (RFLPs), 126 noncoding simple sequence repeats (SSRs), 114 expressed sequence tag polymorphisms (ESTPs), 71 SSRs within ESTs, and 17 presence/absence variants (PAVs) of ESTs (File S7). Of the 3856 markers mapped, 3639 (94%) aligned to *P. taeda* transcript assemblies with an average sequence identity of 98.8%. Predicted functions for 3082 mapped ESTs were obtained through alignment of the translated transcripts to 2385 unique *Arabidopsis* protein sequences in the TAIR10 database and to 1419 unique conserved domains in the NCBI CDD database. A total of 3762 mapped markers aligned to 3305 *P. taeda* version 1.01 genomic scaffolds (Neale *et al.* 2014; Zimin *et al.* 2014) with 99.0% average nucleotide sequence identity. Of the 357 genomic scaffolds that aligned with more than one marker, 168 scaffolds aligned with markers on different LGs and 189 scaffolds aligned with markers from the same LG. Most scaffolds were too short to span multiple markers precluding unbiased estimation of physical to genetic distance.

**Figure 2:**
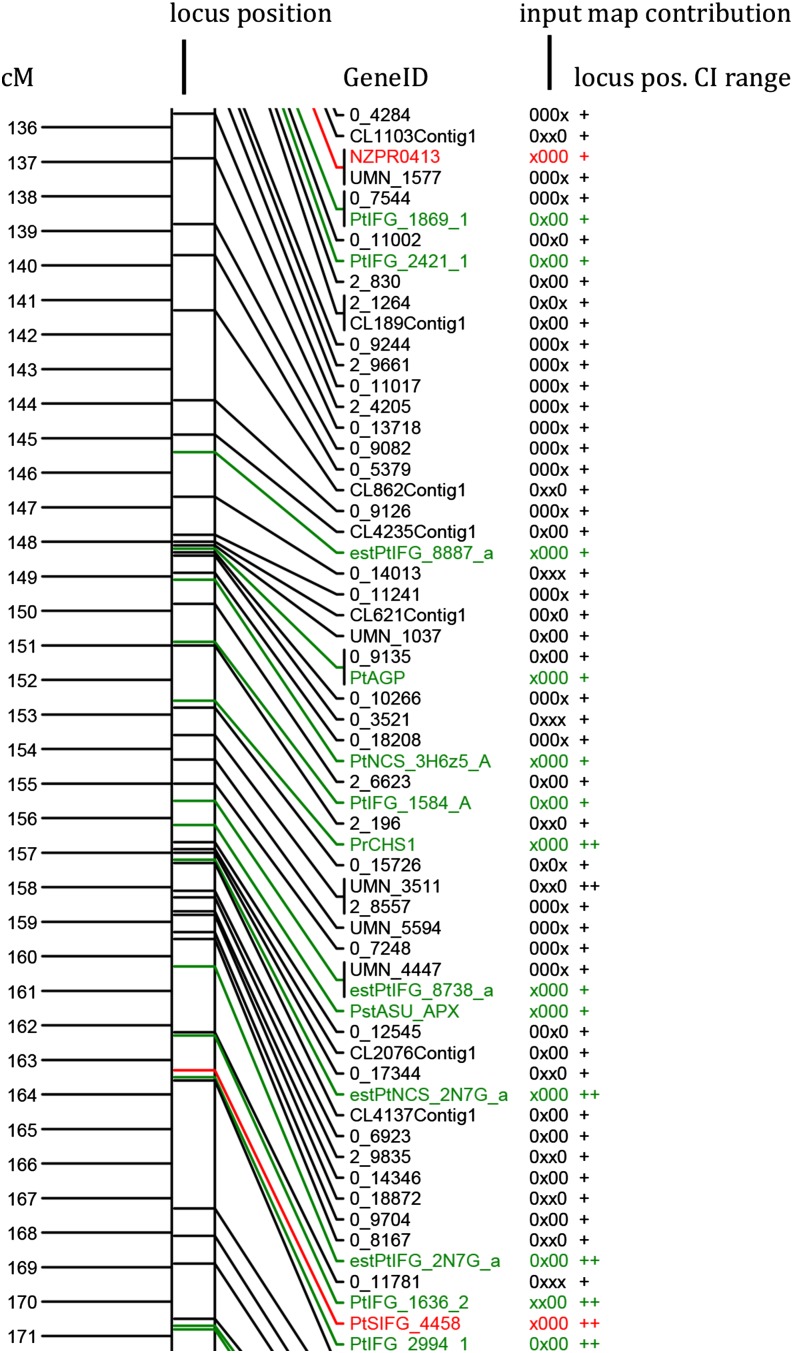
*Pinus taeda* and *Pinus elliottii* consensus map features; centimorgan (cM) scale left of bar, locus mean consensus position along bar; GeneID right of bar; marker type (font color): SNP and PAV (black), SSR (red), ESTP and RFLP (green); the variable next to GeneID indicates whether the locus was present (x) or absent (0) on the four input maps, listed in the following order: QTL-BASE1, QTL-BASE2, BC1, 10-5; far right column denotes cM range of upper and lower bounds of the 95% C.I. for the locus position: 0–1 cM (+) and 1–2 cM (++). Detail shown is from LG-4. For full map graphic, see Figure S9.

Between 275 and 351 markers were positioned on individual linkage groups, and linkage group lengths varied from 147 cM to 222 cM (Table 2). Average and maximum distances between markers were 0.60 cM and 8.47 cM, respectively (Table 2). Average 95% C.I.s for marker positions, estimated over 100 MergeMap runs, varied from 0.15 cM to 1.75 cM for individual LGs and was 0.90 cM genome-wide. Figure 2 displays a segment of the consensus map (Figure S9), which includes marker types, positions, 95% C.I.s of positions, and presence or absence of markers in the input maps.

**Table 2:** Summary of the consensus genetic map for *Pinus taeda and Pinus elliottii* by linkage group (LG)

LG	*N* markers	Length, cM	Average Marker Spacing, cM	Maximum Marker Spacing, cM	Average 95% C.I. Marker Positions, cM
1	305	184.89	0.61	4.94	1.75
2	351	222.00	0.63	5.85	1.22
3	342	186.88	0.55	4.05	0.66
4	306	186.32	0.61	6.77	0.47
5	376	216.41	0.58	6.77	1.55
6	326	193.57	0.60	7.65	0.69
7	304	193.43	0.64	6.42	0.15
8	338	189.56	0.56	4.39	0.57
9	323	172.00	0.53	5.98	0.41
10	331	211.00	0.64	4.20	1.44
11	275	146.89	0.54	5.35	1.07
12	279	202.48	0.73	8.47	0.68
Total	**3856**	**2305.42**			
Average	321	192.12	0.60	5.87	0.90

### Density of markers in the consensus map and in two *P. taeda* genotype-phenotype discovery populations

**Figure 3:**
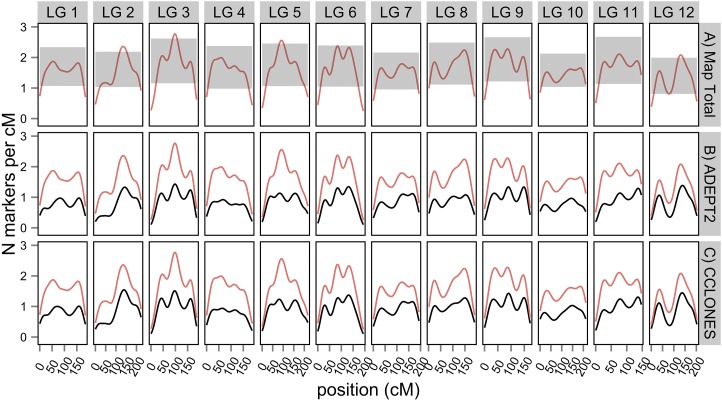
Kernel density estimation of mapped markers in the MergeMap consensus genetic map and in two genotype-phenotype discovery populations of *Pinus taeda*. (A) Marker densities in the consensus map (red lines) were compared to the 95% C.I. of a Poisson distribution (gray regions) of random deviations from uniform marker densities. The densities of SNPs mapped in (B) ADEPT2 (unrelated association) or (C) CCLONES (multiple-family pedigree) populations (black lines) were compared against marker densities in the consensus map (red lines).

Marker density (N cM^−1^) in the consensus map varied from 0.25 to 2.76, with a mean of 1.6 across LGs. Observed variation in the number of markers per cM in the consensus map was compared to random variation in marker density expected from a Poisson distribution (Figure 3A). Lower than expected marker densities were observed toward the distal ends of all 12 LGs, whereas greater than expected marker densities were observed in putative centromeric regions of LGs 2, 3, and 12. The consensus map positioned 2673 of 3938 SNPs (68%) genotyped in unrelated individuals from the *P. taeda* ADEPT2 population and 2829 of 4854 SNPs (58%) in the CCLONES multiple-family pedigree. The density of mapped markers varied from 0.10 to 1.43 in ADEPT2 (mean = 0.85) and from 0.1 to 1.54 in CCLONES (mean = 0.91) (Figure 3, B and C).

### Patterns of linkage disequilibrium in unrelated and pedigreed populations of *P. taeda*

**Figure 4:**
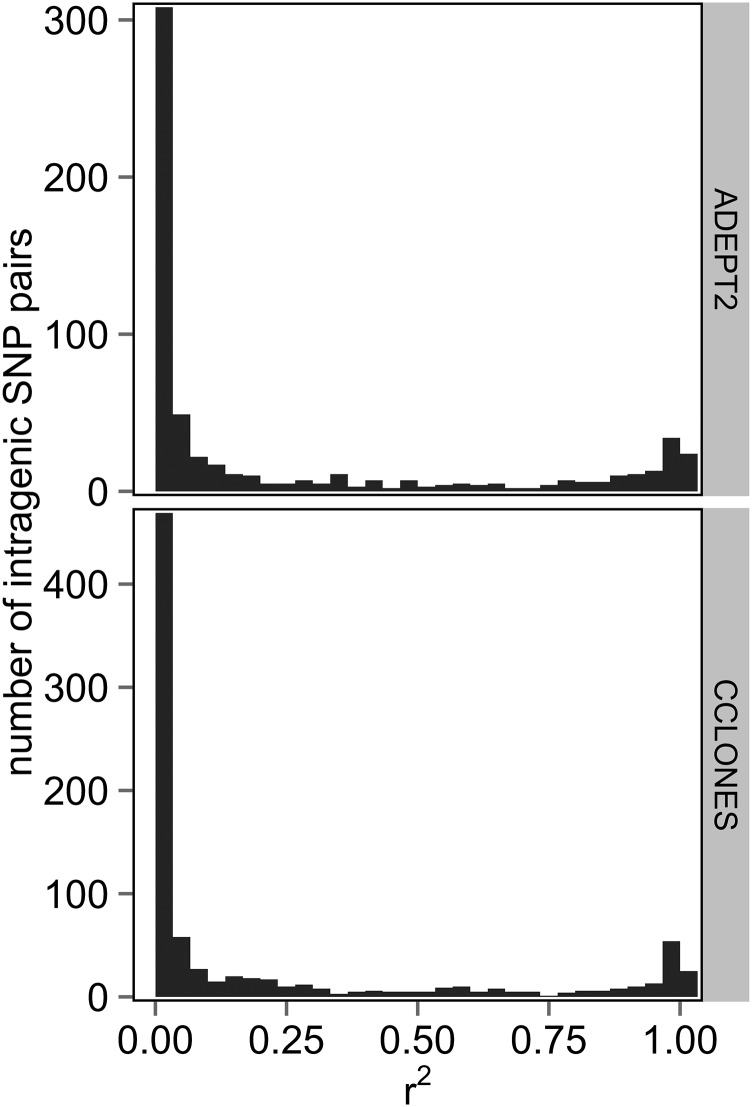
Distributions of r^2^ between pairs of SNP loci within genes in the ADEPT2 (unrelated association) and CCLONES (multiple-family pedigree) populations of *P. taeda*

**Figure 5:**
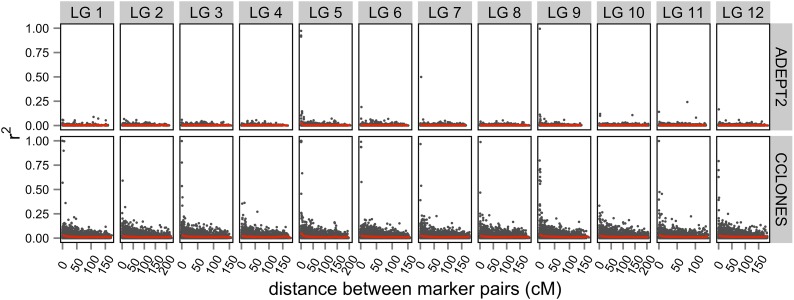
Linkage disequilibrium r^2^ values between loci on the same LGs as a function of genetic distance in ADEPT2 (unrelated association) and CCLONES (multiple-family pedigree) populations of *P. taeda*. Displayed are values of r^2^ between mapped SNPs in different ESTs with minor allele frequencies greater than 0.1 and with less than 50% missing data. Red lines are kernel regressions of r^2^ *vs.* genetic distance.

The r^2^ values were first estimated without accounting for kinship in CCLONES or subpopulation structure in ADEPT2 to compare baseline LD between populations at different scales (*i.e.*, within genes, within LGs, and between LGs). The distributions of r^2^ between SNPs within genes were bimodal in CCLONES and ADEPT2, with a high frequency of r^2^ values that were approximately 0 and a lower frequency of r^2^ values that were approximately 1 (Figure 4). Average r^2^ (±1 SE) between markers separated by less than 1 cM within LGs of the consensus map was 0.027 (±0.001) in ADEPT2 and 0.048 (±0.001) in CCLONES. Percentages of SNP pairs less than 1 cM apart with r^2^ greater than 0.1 varied from 4.2% to 12.6% in ADEPT2 and from 7.3% to 18.1% in CCLONES, depending on minimum minor allele frequency (MAF) thresholds (Table 3). Average LD between markers on the same LGs did not decay substantially with genetic distance in ADEPT2 and CCLONES (Figure 5).

**Table 3:** Number and percentage of SNP pairs among different genes with r^2^ > 0.1 for three linkage classes

	MAF	ADEPT2 Unrelated Association		CCLONES Multiple-Family Pedigree	
		No. (total)	%	No. (total)	%
Within LGs, <1 cM					
Before r^2^ adjustment	0.001	110 (2617)	4.2	210 (2921)	7.2
	0.1	81 (1247)	6.5	134 (1286)	10.4
	0.2	63 (501)	12.6	92 (504)	18.1
After r^2^ adjustment	0.001	103 (2617)	3.9	186 (2921)	6.4
	0.1	79 (1247)	6.3	115 (1284)	9.0
	0.2	58 (501)	11.6	62 (507)	12.2
Within LGs, >1 cM					
Before r^2^ adjustment	0.001	142 (174,032)	<0.1	3498 (198,910)	1.8
	0.1	88 (82,576)	0.1	1274 (85,524)	1.5
	0.2	73 (32,452)	0.2	621 (33,007)	1.9
After r^2^ adjustment	0.001	125 (174,032)	<0.1	1434 (198,910)	0.7
	0.1	80 (82,576)	0.1	412 (85,524)	0.5
	0.2	60 (32,452)	0.2	116 (33,007)	0.4
Between LGs					
Before r^2^ adjustment	0.001	305 (1,933,979)	<0.1	16,999 (2,208,279)	0.8
	0.1	8 (922,248)	<0.1	5256 (953,810)	0.6
	0.2	0 (360,875)	0	2485 (368,881)	0.7
After r^2^ adjustment	0.001	229 (1,933,979)	<0.1	3052 (2208,279)	0.1
	0.1	0 (922,248)	0	558 (953,810)	0.1
	0.2	0 (360,875)	0	370 (368,881)	0.1

Number and percentage of SNP pairs among different genes with r^2^ > 0.1 for three linkage classes before and after adjusting r^2^ for subpopulation structure in ADEPT2 (unrelated) and kinship in CCLONES (multiple-family pedigree). MAF, minimum minor allele frequency threshold for SNP pairs; LG, linkage group.

**Figure 6:**
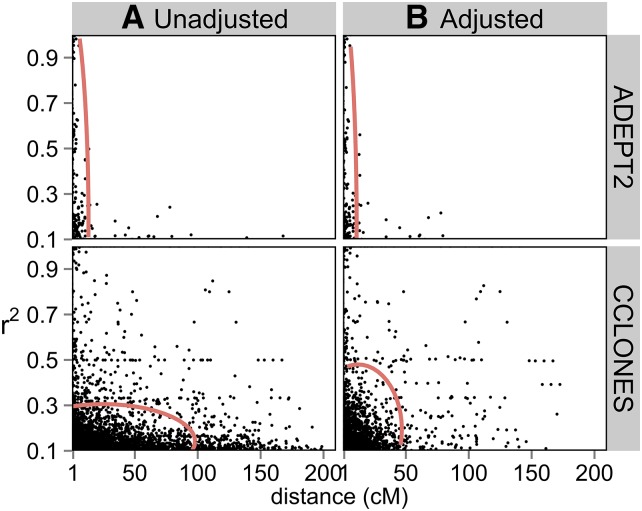
Extended linkage disequilibrium within linkage groups in the ADEPT2 (unrelated association) and CCLONES (multiple-family pedigree) populations of *P. taeda*. Values of r^2^ *vs.* genetic distance are plotted for SNP pairs with r^2^ > 0.1 that were more than 1 cM apart (A) before and (B) after accounting for population structure in ADEPT2 and kinship in CCLONES. The 95% confidence ellipses of r^2^ *vs.* genetic distance are depicted in red.

Extended LD within LGs, defined as SNPs more than 1 cM apart with r^2^ values greater than 0.1, was rare in ADEPT2, occurring between 0.08% and 0.22% of locus pairs (Table 3). Extended LD was more prevalent in CCLONES, occurring between 1.5% and 1.9% of SNP pairs. Furthermore, the range of genetic distances over which extended LD was observed was greater in CCLONES as compared to ADEPT2 (Figure 6). LD between SNPs on different LGs was rare in both populations, but CCLONES had a greater percentage of SNP pairs on different LGs with r^2^ > 0.1 (0.6%–0.8%) as compared to ADEPT2 (<0.1%) (Table 3). Pairs of mapped and unmapped SNPs with r^2^ > 0.1 in ADEPT2 and CCLONES are reported in File S9.

### The effects of subpopulation structure and kinship on linkage disequilibrium

**Figure 7:**
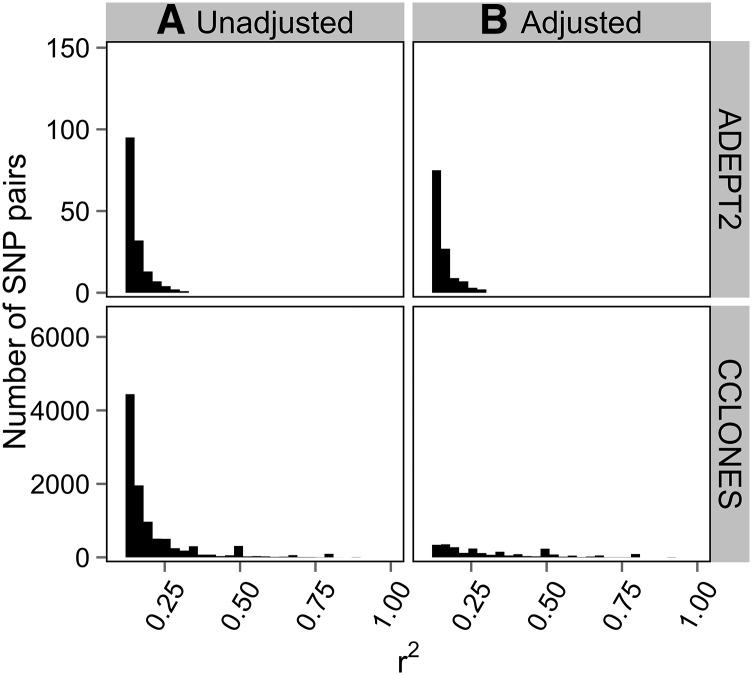
Linkage disequilibrium between SNPs on different linkage groups in the ADEPT2 (unrelated association) and CCLONES (multiple-family pedigree) populations of *P. taeda*. Distributions of r^2^ were plotted for SNP pairs on different LGs with r^2^ > 0.1. The distributions were compared (A) before and (B) after adjusting r^2^ values for subpopulation structure in ADEPT2 and kinship in CCLONES.

Adjusting r^2^ for structure in ADEPT2 or kinship in CCLONES reduced number of locus pairs with r^2^ > 0.1 in both populations (Table 3). Larger reductions in the extent of LD within LGs (Figure 6) and the percentages of SNPs in LD between LGs (Figure 7) were observed after adjusting for kinship in CCLONES as compared to adjusting for structure in ADEPT2. Some cases of extended LD within LGs may have been attributable to error in the estimation of marker positions or spurious effects due to low minor allele frequency (Plomion *et al.* 2014). Considering only pairs of SNPs where both loci were separated by more than 5 cM within a single input map, and considering that both SNPs had MAF >0.1, 135 SNP pairs in CCLONES and seven SNP pairs in ADEPT2 had adjusted r^2^ > 0.1. No SNP pairs on different LGs had adjusted r^2^ > 0.1 in ADEPT2, and 558 SNP pairs had adjusted r^2^ > 0.1 in CCLONES (MAF >0.1) (Table 3). To test for possible epistatic LD between SNPs on different LGs in CCLONES, the distribution of adjusted r^2^ values from 135 SNP pairs with strong evidence for extended LD within LGs was used to estimate a critical value of r^2^ > 0.82 that exceeded the Bonferroni significance threshold (α = 0.05/558 tests). No locus pairs on different LGs had adjusted r^2^ that exceeded the 0.82 significance threshold in CCLONES (Figure 7).

## Discussion

The *Pinus taeda* and *Pinus elliotti* consensus map positioned 3555 polymorphic transcripts and 301 noncoding markers that segregated in four mapping populations composed of 1251 individuals. The consensus map positioned 1.3- to 8.4-times the number of markers of the input maps (Table 1) and genetically mapped approximately 7% of the 50,172 genes predicted for *P. taeda* (Neale *et al.* 2014). Alignment of markers in the consensus map to the *Pinus taeda* genome assembly version 1.01 (Neale *et al.* 2014; Zimin *et al.* 2014) positioned 3305 scaffolds onto LGs. Improvement in the functional predictions for 3082 mapped genes was attained by aligning partial length ESTs (File S5) to longer *P. taeda* transcript assemblies.

Colinearity among the QTL-BASE1, QTL-BASE2, and BC1 input maps, in contrast with large marker order reversals on five linkage groups in the original 10-5 map (Neves *et al.* 2014), indicated that the order reversals in the 10-5 map were likely to be mapping errors (Figure S1, Figure S2, Figure S3, and Figure S4). This interpretation was supported by the strong synteny of homologous markers among conifer genetic maps (Brown *et al.* 2001; Krutovsky *et al.* 2004; Pavy *et al.* 2012b) and the fact that the 10-5 map was constructed from a much smaller population of 72 haploid megagametophytes (Table 1). Reconstruction of the 10-5 map by specifying subsets of fixed and start order loci and removing loci with suspect linkages produced a map that was collinear with the other input maps (Figure S6 and Figure S7) and the consensus map (Figure 1).

Marker density in the consensus map varied from less than one marker per cM toward the distal ends of LGs to three markers per cM toward the middle of LGs (Figure 3). Similar patterns of marker densities have been observed in the consensus genetic maps of *Picea glauca* (white spruce) and *Picea mariana* (black spruce) and *Pinus pinaster* (maritime pine) (Pavy *et al.* 2012b; Plomion *et al.* 2014). Regions of high marker density may occur in centromeric regions with reduced recombination rates, whereas regions of low marker density are associated with telomeres with higher rates of recombination. Power to detect associations between markers and traits is greater in regions with reduced recombination rates, but the genomic resolution to fine-map causal variants in these regions is reduced (Nachman 2002).

### Comparison of LD extent between discovery populations: implications for comparative QTL and association mapping across populations

LD was expected to be rare and to decay rapidly among distantly related individuals in ADEPT2, whereas pedigree relationships in CCLONES were expected to increase the extent of LD. Extended LD within LGs and LD between SNPs (Figure 6) on different LGs (Figure 7) was more prevalent in CCLONES compared to ADEPT2 (Table 3). Greater reductions in the prevalence and extent of LD within LGs and between LGs after accounting for kinship in CCLONES *vs.* structure in ADEPT2 (Table 3) suggest that pedigree relationships increased LD more than population structure in these populations. The relatively small effects of structure on patterns of LD may be explained by high rates of gene flow and weak subpopulation structure across the geographic range of *P. taeda* (Al-Rabab’ah and Williams 2002; Eckert *et al.* 2010; Chhatre *et al.* 2013).

LD between SNPs within the same gene (Figure 4) and between SNPs at different loci on the same LGs tended to be weak in both populations (Table 3), and LD did not decay substantially with genetic distance (Figure 5). After adjusting r^2^ values for kinship and structure and after accounting for uncertainty marker position, cases of extended LD within LGs were rare. There was no evidence for epistatic LD between SNPs on different LGs in either population. Low levels of LD in CCLONES may be explained by the fact that 80% of the pairs of the individuals within the population were unrelated (Table S2).

Low average r^2^ between SNPs on the same LG and the lack of decay of r^2^ with genetic distance imply that association genetic studies in these populations are underpowered to comprehensively detect causal variants at current marker densities. This result is not surprising considering that LD decays within hundreds to thousands of bases in outcrossing *P. taeda* populations (Brown *et al.* 2004; Neale and Savolainen 2004). Despite the low levels of LD observed in CCLONES, markers genotyped at low densities may be predictive for genomic selection in complex pedigrees of *P. taeda*. The 4854 SNP loci currently genotyped in CCLONES had substantial predictive abilities for traits related to growth, development, wood quality, disease resistance, and insect resistance (Resende *et al.* 2012b; Westbrook *et al.* 2013; Westbrook *et al.* 2015). The predictive abilities of these markers coupled with the low levels of LD in CCLONES suggest that a large proportion of the predictive ability of low-density marker panels is derived from tracing pedigree relationships rather than being tightly linked to causal polymorphisms (Wientjes *et al.* 2013).

## Conclusions

The consensus genetic map for *P. taeda* and *P. elliottii* presented here is the most densely populated linkage map for a conifer to date (Ritland *et al.* 2011). The consensus map coupled with the genome-wide analysis of linkage disequilibrium in two discovery populations of *Pinus taeda* establishes a foundation for comparative association mapping between populations and the implementation of genomic selection in loblolly pine and slash pine.

## 

## Supplementary Material

Supporting Information

## References

[bib1] Al-Rabab’ah M. A. Williams C. G., 2002 Population dynamics of *Pinus taeda* L. based on nuclear microsatellites. For. Ecol. Manage. 163: 263–271.

[bib2] Altschul S. F. Gish W. Miller W. Myers E. W. Lipman D. J., 1990 Basic Local Alignment Search Tool. J. Mol. Biol. 215: 403–410. 2231712 10.1016/S0022-2836(05)80360-2

[bib3] Baltunis B. S. Huber D. A. White T. L. Goldfarb B. Stelzer H. E., 2007 Genetic analysis of early field growth of loblolly pine clones and seedlings from the same full-sib families. Can. J. For. Res. 37: 195–205.

[bib4] Boratyn G. M. Schäffer A. A. Agarwala R. Atschul S. F. Lipman D. J., 2012 Domain enhanced lookup time accelerated BLAST. Biol. Direct 7: 12. 22510480 10.1186/1745-6150-7-12 PMC3438057

[bib5] Brown G. R. Gill G. P. Kuntz R. J. Langley C. H. Neale D. B., 2004 Nucleotide diversity and linkage disequilibrium in loblolly pine. Proc. Natl. Acad. Sci. USA 101: 15255–15260. 15477602 10.1073/pnas.0404231101 PMC524047

[bib6] Brown G. R. Kadel E. E. Bassoni D. L. Kiehne K. L. Temesgen B., 2001 Anchored reference loci in loblolly pine (*Pinus taeda* L.) for integrating pine genomics. Genetics 159: 799–809. 11606554 10.1093/genetics/159.2.799 PMC1461821

[bib7] Chhatre V. E. Byram T. D. Neale D. B. Wegrzyn J. L. Krutovsky K. V., 2013 Genetic structure and association mapping of adaptive and selective traits in the east Texas loblolly pine (*Pinus taeda* L.) breeding populations. Tree Genet. Genomes 9: 1161–1178.

[bib8] Cumbie W. P. Eckert A. J. Wegrzyn J. L. Whetten R. Neale D. B., 2011 Association genetics of carbon isotope discrimination, height and foliar nitrogen in a natural population of *Pinus taeda* L. Heredity 107: 105–114. 21245892 10.1038/hdy.2010.168 PMC3178403

[bib9] Echt C. S. Saha S. Krutovsky K. Wimalanathan K. Erpelding J., 2011 An annotated genetic map of loblolly pine based on microsatellite and DNA markers. BMC Genet. 12: 17. 21269494 10.1186/1471-2156-12-17 PMC3038140

[bib10] Eckert A. J. van Heerwaarden J. Wegrzyn J. L. Nelson C. D. Ross–Ibarra J., 2010 Patterns of population structure and environmental associations to aridity across the range of loblolly pine (*Pinus taeda* L., Pinaceae). Genetics 185: 969–982. 20439779 10.1534/genetics.110.115543 PMC2907212

[bib11] Endelman J. Plomion C., 2014 LPmerge: An R package for merging genetic maps by linear programming. Bioinformatics 30: 1623–1624. 24532720 10.1093/bioinformatics/btu091

[bib12] Flint-Garcia S. A. Thornsberry J. M. Buckler E. S., 2003 Structure of linkage disequilibrium in plants. Annu. Rev. Plant Biol. 54: 357–374. 14502995 10.1146/annurev.arplant.54.031902.134907

[bib14] Gaut B. S. Long A. D., 2003 The Lowdown of Linkage Disequilibrium. Plant Cell 15: 1502–1506. 12837942 10.1105/tpc.150730 PMC526043

[bib15] Habier D. Fernando R. L. Dekkers J. C. M., 2009 Genomic selection using low–density panels. Genetics 182: 343–353. 19299339 10.1534/genetics.108.100289 PMC2674831

[bib16] Henderson C. R., 1976 A simple method for computing the inverse of a numerator relationship matrix used in the prediction of breeding values. Biometrics 32: 69–83.

[bib17] Krutovsky K. Troggio M. Brown G. Jermstad K. Neale D., 2004 Comparative mapping in the Pinaceae. Genetics 168: 447–461. 15454556 10.1534/genetics.104.028381 PMC1448108

[bib18] Lamesch P. Berardini T. Z. Li D. Swarbrek D. Wilks C., 2011 The Arabidopsis Information Resource (TAIR): improved gene annotation and new tools. Nucleic Acids Res. 40: D1202–D1210. 22140109 10.1093/nar/gkr1090 PMC3245047

[bib19] Maliepaard C. Jansen J. Van Ooijen J. W., 1997 Linkage analysis in a full–sib family of an outbreeding plant species: overview and consequences for applications. Genet. Res. 70: 237–250.

[bib20] Mangin B. Siberchicot A. Nicolas S. Doligez A. This P., 2012 Novel measures of linkage disequilibrium that correct the bias due to population structure and relatedness. Heredity 108: 285–291. 21878986 10.1038/hdy.2011.73 PMC3282397

[bib21] Marchler-Bauer A. Zheng C. Chitaz F. Derbyshire M. Geer L., 2013 CDD: conserved domains and protein three-dimensional structure. Nucleic Acids Res. 41: D348–D352. 23197659 10.1093/nar/gks1243 PMC3531192

[bib22] Martínez-García P. Stevens K. Wegrzyn J. Liechty J. Crepeau M., 2013 Combination of multipoint maximum likelihood (MML) and regression mapping algorithms to construct a high–density genetic linkage map for loblolly pine (*Pinus taeda* L.). Tree Genet. Genomes 9: 1529–1535.

[bib23] McKeand S. Mullin T. Byram T. White T., 2003 Deployment of genetically improved loblolly and slash pines in the South. J. For. 101: 32–37.

[bib24] Meuwissen T. H. E. Hayes B. J. Goddard M. E., 2001 Prediction of total genetic value using genome–wide dense marker maps. Genetics 157: 1819–1829. 11290733 10.1093/genetics/157.4.1819 PMC1461589

[bib25] Muñoz P. R. Huber D. A. Butnor J. R., 2011 Phenotypic analysis of first–year traits in a pseudo–backcross {(slash x loblolly) x slash} and the open–pollinated families of the pure–species progenitors. Tree Genet. Genomes 7: 183–192.

[bib26] Nachman M. W., 2002 Variation in recombination rate across the genome: Evidence and implications. Curr. Opin. Genet. Dev. 12: 657–663. 12433578 10.1016/s0959-437x(02)00358-1

[bib27] Neale D. Wegrzyn J. Stevens K. Zimin A. Puiu D., 2014 Decoding the massive genome of loblolly pine using haploid DNA and novel assembly strategies. Genome Biol. 15: R59. 24647006 10.1186/gb-2014-15-3-r59 PMC4053751

[bib28] Neale D. B. Savolainen O., 2004 Association genetics of complex traits in conifers. Trends Plant Sci. 9: 325–330. 15231277 10.1016/j.tplants.2004.05.006

[bib29] Neves, L. G., J. M. Davis, W. B. Barbazuk, and M. Kirst, 2014 A high-density gene map of loblolly pine (*Pinus taeda* L.) based on exome sequence capture genotyping. G3 (Bethesda) 4: 29–37. 10.1534/g3.113.008714 PMC3887537 24192835

[bib30] Pavy N. Namroud M.-C. Gagnon F. Isabel N. Bosquet J., 2012a The heterogeneous levels of linkage disequilibrium in white spruce genes and comparative analysis with other conifers. Heredity 108: 273–284. 21897435 10.1038/hdy.2011.72 PMC3282396

[bib31] Pavy N. Pelgas B. Laroche J. Rigault P. Isabel N., 2012b A spruce gene map infers ancient plant genome reshuffling and subsequent slow evolution in the gymnosperm lineage leading to extant conifers. BMC Biol. 10: 84. 23102090 10.1186/1741-7007-10-84 PMC3519789

[bib32] Platt A. Vilhjálmsson B. J. Nordborg M., 2010 Conditions under which genome–wide association studies will be positively misleading. Genetics 186: 1045–1052. 20813880 10.1534/genetics.110.121665 PMC2975277

[bib33] Plomion C. Chancerel E. Endelman J. Lamy J.-B. Mandrou E., 2014 Genome–wide distribution of genetic diversity and linkage disequilibrium in a mass–selected population of maritime pine. BMC Genomics 15: 171. 24581176 10.1186/1471-2164-15-171 PMC4029062

[bib34] Prestemon, J. P., and R. C. Abt, 2002 TIMBR-1: Timber products supply and demand, pp. 299–325 in *Southern Forest Resource Assessment*. Southern Research Station General Technical Report SRS–53, Asheville, NC.

[bib35] Pritchard J. K. Przeworski M., 2001 Linkage disequilibrium in humans: Models and data. Am. J. Hum. Genet. 69: 1–14. 11410837 10.1086/321275 PMC1226024

[bib36] Quesada T. Gopal V. Cumbie W. P. Eckert A. J. Wegrzyn J. L., 2010 Association mapping of quantitative disease resistance in a natural population of loblolly pine (*Pinus taeda* L.). Genetics 186: 677–686. 20628037 10.1534/genetics.110.117549 PMC2954465

[bib37] R Core Team, 2012 R: Language and environment for statistical computing. R Foundation for Statistical Computing, Vienna, Austria.

[bib38] Raj A. Stephens M. Pritchard J. K., 2014 fastSTRUCTURE: variational inference of population structure in large SNP datasets. Genetics 197: 573–589. 24700103 10.1534/genetics.114.164350 PMC4063916

[bib39] Resende M. F. R. Muñoz P. Acosta J. J. Peter G. F. Davis J. M., 2012a Accelerating the domestication of trees using genomic selection: accuracy of prediction models across ages and environments. New Phytol. 193: 617–624. 21973055 10.1111/j.1469-8137.2011.03895.x

[bib40] Resende M. F. R. Munoz P. Resende M. D. V. Garrick D. J. Fernando R. L., 2012b Accuracy of genomic selection methods in a standard data set of loblolly pine (*Pinus taeda* L.). Genetics 190: 1503. 22271763 10.1534/genetics.111.137026 PMC3316659

[bib41] Ritland K. Krutovsky K. V. Tsumura Y. Pelgas B. Isabel N., 2011 Genetic mapping in conifers, pp. 196–238 in *Genetics*, *Genomics and Breeding of Conifers*, edited by Plomion C. Bousquet J. Kole C. Science Publisher, Enfield, Massachusetts.

[bib42] Silverman B. W., 1986 Density Estimation for Statistics and Data Analysis, Chapman and Hall, London.

[bib43] Smith W. B. Miles P. D. Perry C. H. Pugh S. A., 2007 Forest resources of the United States: a technical document supporting the Forest Service 2010 RPA assessment. US Department of Agriculture, Forest Service, Washington Office, Washington, DC.

[bib44] Van Ooijen J. W., 2011 JoinMap 4.1, Software for the Calculation of Genetic Linkage Maps, Plant Research International, Wageningen, the Netherlands.

[bib45] Wegrzyn J. Liechty J. Stevens K. Wu L. Loopstra C., 2014 Unique features of the loblolly pine (*Pinus taeda* L.) megagenome revealed through sequence annotation. Genetics 196: 891–909. 24653211 10.1534/genetics.113.159996 PMC3948814

[bib46] Wenzl P. Li H. Carling J. Zhou M. Raman H., 2006 A high–density consensus map of barley linking DArT markers to SSR, RFLP, and STS loci and agricultural traits. BMC Genomics 7: 206. 16904008 10.1186/1471-2164-7-206 PMC1564146

[bib47] Westbrook J. W. Resende M. F. R. Muñoz P. Walker A. R. Wegrzyn J. L., 2013 Association genetics of oleoresin flow in loblolly pine: discovering genes and predicting phenotype for improved resistance to bark beetles and bioenergy potential. New Phytol. 199: 89–100. 23534834 10.1111/nph.12240

[bib48] Westbrook J. W. Walker A. R. Neves L. G. Muñoz P. Resende M. F. R., 2015 Discovering candidate genes that regulate resin canal number in *Pinus taeda* stems by integrating association genetics and QTL analysis across environments, ages, and populations. New Phytol. 205: 627–641. 25266813 10.1111/nph.13074

[bib49] Wickham, H., 2009 ggplot2: elegant graphics for data analysis. Springer, New York. Available at: http://had.co.nz/ggplot2/book

[bib50] Wientjes Y. C. J. Veerkamp R. F. Calus M. P. L., 2013 The effect of linkage disequilibrium and family relationships on the reliability of genomic prediction. Genetics 193: 621–631. 23267052 10.1534/genetics.112.146290 PMC3567749

[bib51] Wu T. Watanabe C., 2005 GMAP: a genomic mapping and alignment program for mRNA and EST sequences. Bioinformatics 21: 1859–1875. 15728110 10.1093/bioinformatics/bti310

[bib52] Wu Y. Close T. J. Lonardi S., 2011 Accurate construction of consensus genetic maps via integer linear programming. IEEE/ACM Tran. Computational Biol. Bioinformatics 8: 381–394 (TCBB). 10.1109/TCBB.2010.35 20479505

[bib53] Yan J. Shah T. Warburton M. L. Buckler E. S. McMullen M. D., 2009 Genetic characterization and linkage disequilibrium estimation of a global maize collection using SNP markers. PLoS ONE 4: e8451. 20041112 10.1371/journal.pone.0008451 PMC2795174

[bib54] Zimin A. Stevens K. A. Crepeau M. W. Holtz-Morris A. Koriabine M., 2014 Sequencing and assembly of the 22-Gb loblolly pine genome. Genetics 196: 875–890. 24653210 10.1534/genetics.113.159715 PMC3948813

