# Supplementary material for: A Consensus Genetic Map for Pinus taeda and Pinus elliottii and Extent of Linkage Disequilibrium in Two Genotype-Phenotype Discovery Populations of Pinus taeda
Source: G3 (Bethesda). 2015 Jun 11;5(8):1685–94. doi: 10.1534/g3.115.019588 (PMC4528325; doi:10.1534/g3.115.019588)
Supplement: Supporting Information [file supp_5_8_1685__index.html]

A Consensus Genetic Map for Pinus taeda and Pinus elliottii and Extent of Linkage Disequilibrium in Two Genotype-Phenotype Discovery Populations of Pinus taeda — Supporting Information 

# A Consensus Genetic Map for *Pinus taeda* and *Pinus elliottii* and Extent of Linkage Disequilibrium in Two Genotype-Phenotype Discovery Populations of *Pinus taeda*

## Supporting Information for Westbrook *et al.*, 2015

**Files in this Data Supplement:**

- Supporting Information - Figures S1-S9, Tables S1-S5, and Files S1-S9 (PDF, 659 KB)
- Figure S1 - Comparison of order of shared markers between the original 10-5 from Neves *et al.*, (2014) and BC1 input maps. (PDF, 197 KB)
- Figure S2 - Comparison of order of shared markers between the QTL-BASE2 input map and the original 10-5 from Neves *et al.*, (2014). (PDF, 204 KB)
- Figure S3 - Comparison of order of shared markers between the QTL-BASE2 and BC1 input maps. (PDF, 237 KB)
- Figure S4 - Comparison of order of shared markers between the QTL-BASE1 and QTL-BASE2 input maps. (PDF, 218 KB)
- Figure S5 - Population structure analysis in ADEPT2 (a), CCLONES (b) with fastSTRUCTURE (Raj *et al.* 2014). (PDF, 151 KB)
- Figure S6 - Comparison of order of shared markers between the BC1 and reconstructed 10-5 input maps. (PDF, 170 KB)
- Figure S7 - Comparison of order of shared markers between the QTL-BASE2 and reconstructed 10-5 input maps. (PDF, 170 KB)
- Figure S8 - Comparison of order of shared markers between the MergeMap and LPMerge consensus genetic maps for *P. taeda* and *P. elliotti*. (PDF, 245 KB)
- Figure S9 - MapChart of MergeMap consensus genetic map for *P. taeda* and *P. elliottii*. (PDF, 342 KB)
- Table S1 - Summary of reconstructed 10-5 input map, locus counts by linkage group and final map goodness-of-fit chi-square. (PDF, 109 KB)
- Table S2 - Summary of identity by descent (IBD) proportions among pairs of individuals in the CCLONES pedigree. (PDF, 136 KB)
- Table S3 - Number of markers included and shared among input maps used to construct the *P. taeda* consensus map. (PDF, 172 KB)
- Table S4 - Comparisons of the MergeMap and LPmerge *P. taeda* consensus genetic maps. (PDF, 172 KB)
- Table S5 - Root mean squared error (RMSE) in marker order between the MergeMap or LPmerge consensus maps and the input maps by linkage group (LG). (PDF, 150 KB)
- File S1 - The QTL-BASE1 linkage map with GIC. (.txt, 43 KB)
- File S2 - The QTL-BASE2 linkage map with GIC. (.txt, 180 KB)
- File S3 - The BC1 linkage map with GIC. (.txt, 86 KB)
- File S4 - The 10-5 linkage map with GIC. (.txt, 95 KB)
- File S5 - FASTA formatted expressed sequence tag sequences of mapped markers. (.txt, 3 MB)
- File S6 - fastSTRUCTURE results matrix of the ADEPT2 population for three subpopulations. (.txt, 13 KB)
- File S7 - MergeMap consensus linkage map for *Pinus taeda* with alignments to the *P. taeda* genome and transcriptome. (.txt, 1 MB)
- File S8 - LPmerge consensus linkage map for *Pinus taeda* and *Pinus elliotti*. (.txt, 123 KB)
- File S9 - Pairs of expressed sequences containing SNPs in extended LD (R2 >0.1), before and after accounting for structure in ADEPT2 and kinship in CCLONES; table includes MAF and consensus map positions. (.zip, 2 MB)
