# Supplementary material for: A Consensus Genetic Map for Pinus taeda and Pinus elliottii and Extent of Linkage Disequilibrium in Two Genotype-Phenotype Discovery Populations of Pinus taeda
Source: G3 (Bethesda). 2015 Jun 11;5(8):1685–94. doi: 10.1534/g3.115.019588 (PMC4528325; doi:10.1534/g3.115.019588)
Supplement: Supporting Information [file supp_g3.115.019588_019588SI.pdf]

G3· Genes | Genomes | Genetics

A consensus genetic map for *Pinus taeda* and *Pinus elliottii* and extent of linkage disequilibrium in two genotype-phenotype discovery populations of *P. taeda*

Jared W. Westbrook,[*,1] Vikram E. Chhatre,[§,1,2] Le-Shin Wu,[†] Srikar Chamala,[‡, **, §§] Leandro Gomide Neves,[††] Patricio Muñoz,[‡], Pedro J. Martínez-García,[***] David B. Neale,[***] Matias Kirst,[*] Keithanne Mockaitis,[§§§] C. Dana Nelson,[§] Gary F. Peter,[*] John M. Davis,[*] and Craig S. Echt[§]

[*]School of Forest Resources and Conservation, University of Florida, Gainesville, Florida, 32611-0410

[§]Southern Research Station, USDA Forest Service, Saucier, Mississippi, 39574

[†]National Center for Genome Analysis Support, Indiana University, Bloomington, IN 47408

[‡]Department of Biology, University of Florida, Gainesville, FL 32611

[**]Department of Pathology, Immunology and Laboratory Medicine, University of Florida, Gainesville, FL 32603

[§§]Genetics Institute, University of Florida, Gainesville, FL 32608

[††]RAPiD Genomics LLC, Gainesville, FL, 32601

[‡‡]Department of Agronomy, University of Florida, Gainesville, FL 32608

[***]Department of Plant Sciences, University of California, Davis, Davis, CA, 95616

[§§§] Pervasive Technology Institute, Indiana University, Bloomington, IN 47408

[1]These authors contributed equally to this work.

[2]Present address: Department of Plant Biology, University of Vermont, Burlington VT 05405

DOI: 10.1534/g3.115.019588

Supplementary Information

Figure S1 – Comparison of order of shared markers between the original 10-5 from Neves et al., (2014) and BC1 input maps

Figure S2 – Comparison of order of shared markers between the QTL-BASE2 input map and the original 10-5 from Neves *et al*.

Figure S3 - Comparison of order of shared markers between the QTL-BASE2 and BC1 input maps

Figure S4 – Comparison of order of shared markers between the QTL-BASE1 and QTL-BASE2 input maps

Figure S5 - Population structure analysis in ADEPT2 and CCLONES with fastSTRUCTURE

Figure S6 – Comparison of order of shared markers between the BC1 and reconstructed 10-5 input maps

Figure S7 – Comparison of order of shared markers between the QTL-BASE2 and reconstructed 10-5 input maps

Figure S8 – Comparison of order of shared markers between the MergeMap and LPMerge consensus genetic maps for *P. taeda* and *P. elliottii*

Figure S9 – MapChart of MergeMap consensus genetic map for *P. taeda* and *P. elliottii*

Table S1 – Summary of reconstructed 10-5 input map, locus counts by linkage group and final map goodness-of-fit chi-square

Table S2 – Summary of identity by descent (IBD) proportions among pairs of individuals in the CCLONES pedigree

Table S3 – Number of markers included and shared among input maps used to construct the *P. taeda* consensus map

Table S4 – Comparisons of MergeMap and LPmerge *P. taeda* consensus genetic maps

Table S5 - Root mean squared error in marker order between the MergeMap or LPmerge consensus maps and the input maps by linkage group

Supplementary Figures (S1 – S8)

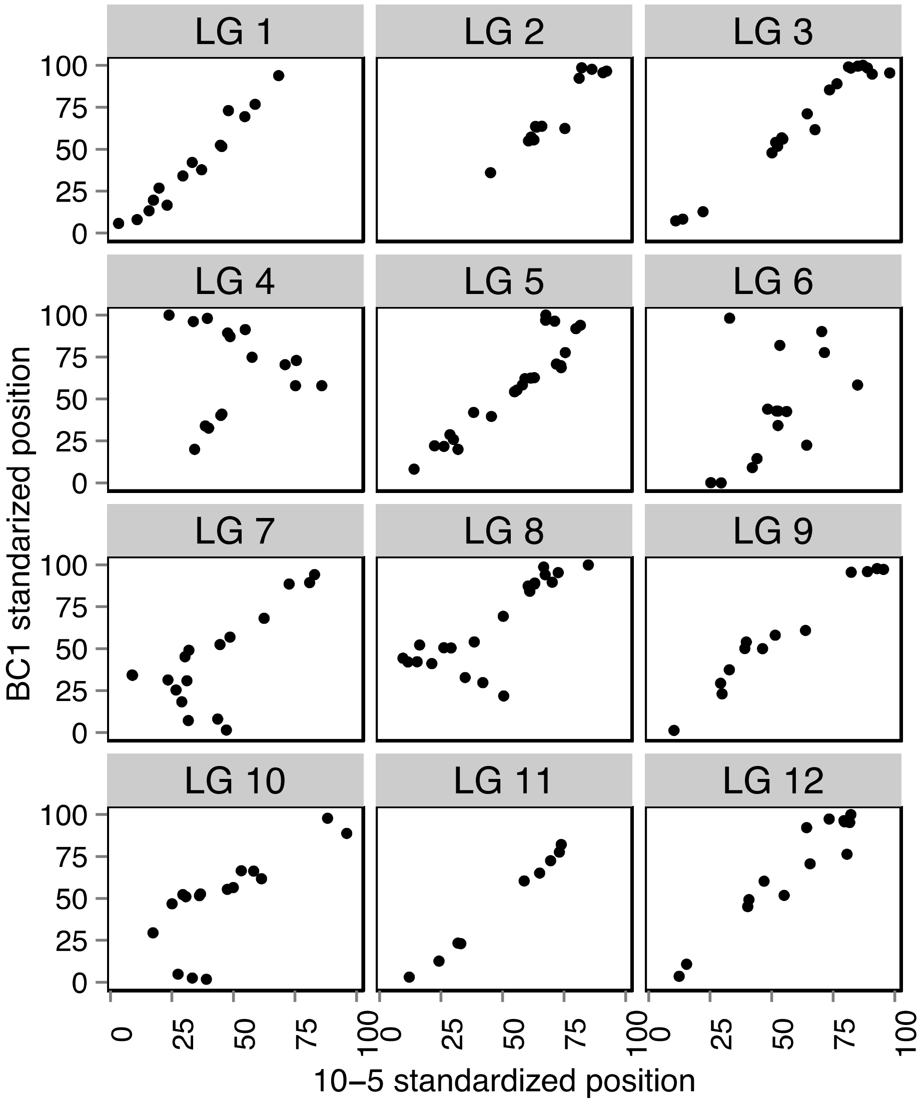

Figure S1 Comparison of order of shared markers between the original 10-5 from Neves *et al.*, (2014) and BC1 input maps. Linkage group lengths were standardized to 100 units for comparison between maps.

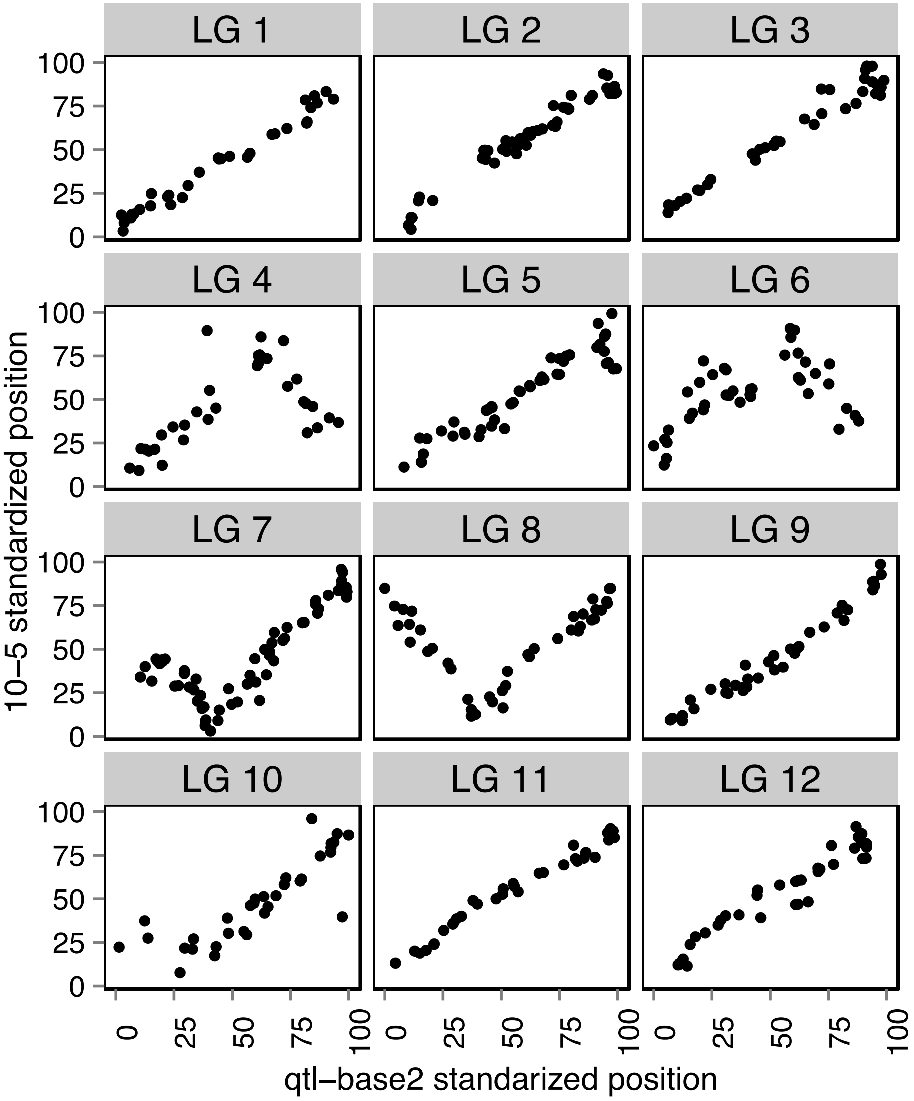

Figure S2 Comparison of order of shared markers between the QTL-BASE2 input map and the original 10-5 from Neves *et al.*, (2014). Linkage group lengths were standardized to 100 units for comparison between maps.

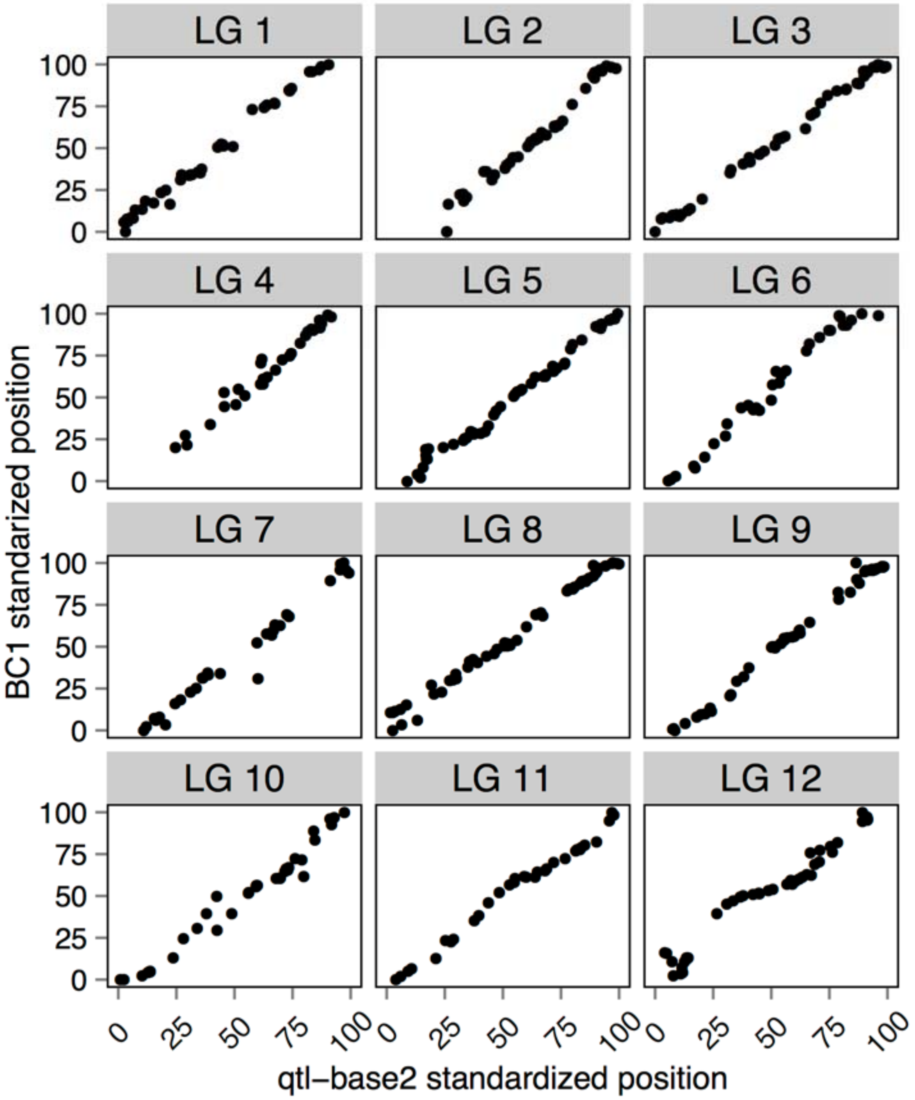

Figure S3 Comparison of order of shared markers between the QTL-BASE2 and BC1 input maps. Linkage group lengths were standardized to 100 units for comparison between maps.

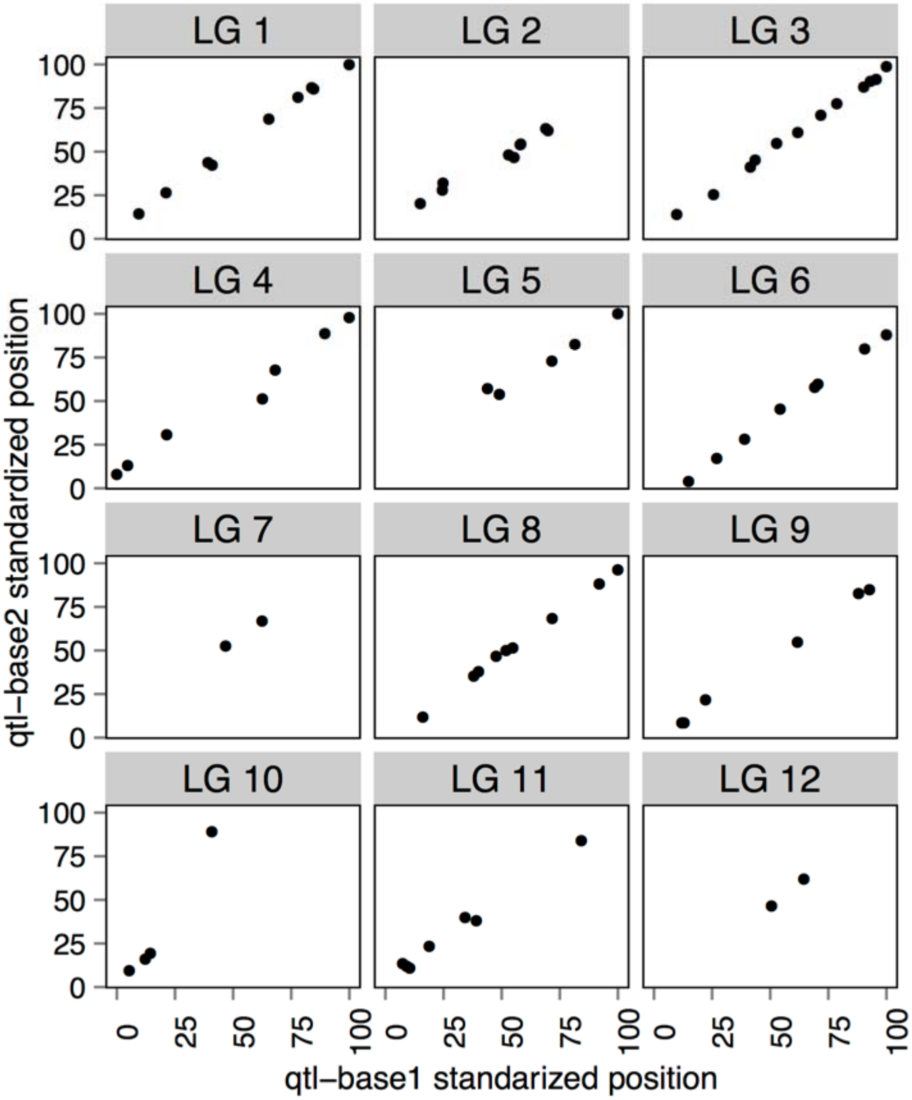

Figure S4 Comparison of order of shared markers between the QTL-BASE1 and QTL-BASE2 input maps. Linkage group lengths were standardized to 100 units for comparison between maps.

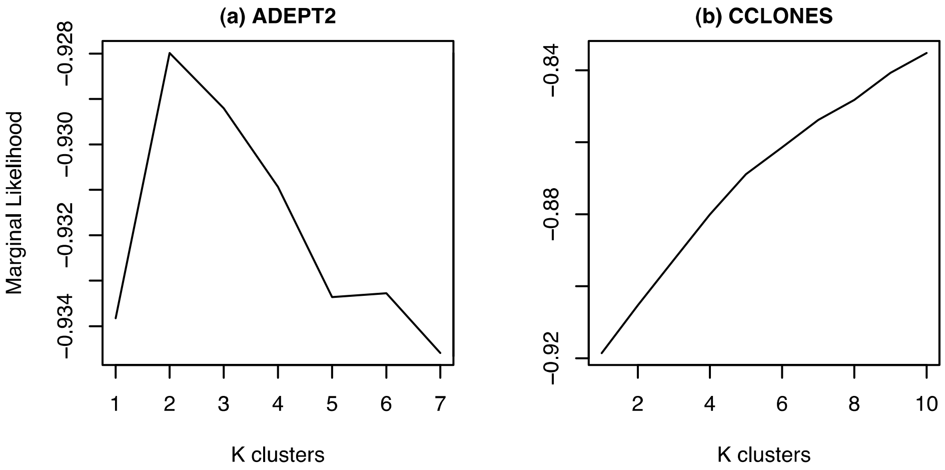

Figure S5 Population structure analysis in ADEPT2 (a), CCLONES (b) with fastSTRUCTURE (Raj et al. 2014). Lines show marginal likelihood at each K

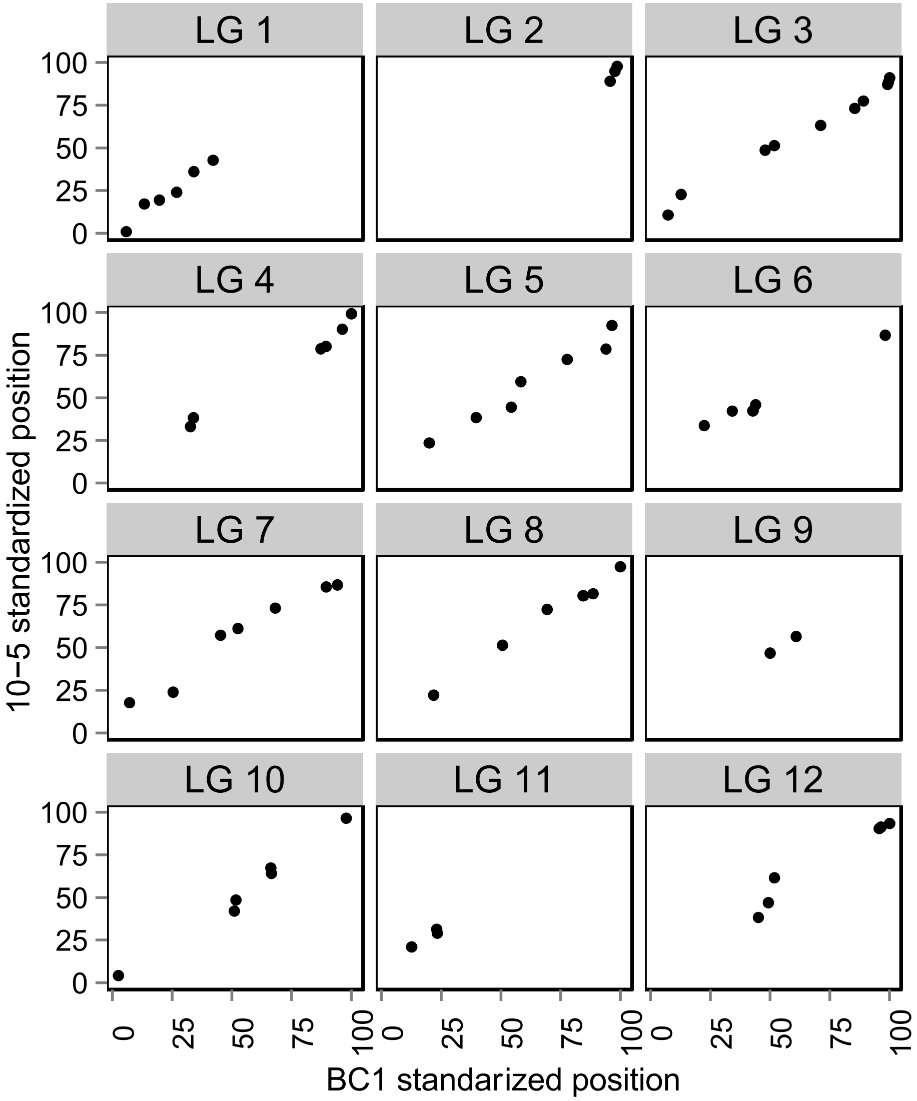

Figure S6 Comparison of order of shared markers between the BC1 and reconstructed 10-5 input maps. Linkage group lengths were standardized to 100 units for comparison between maps.

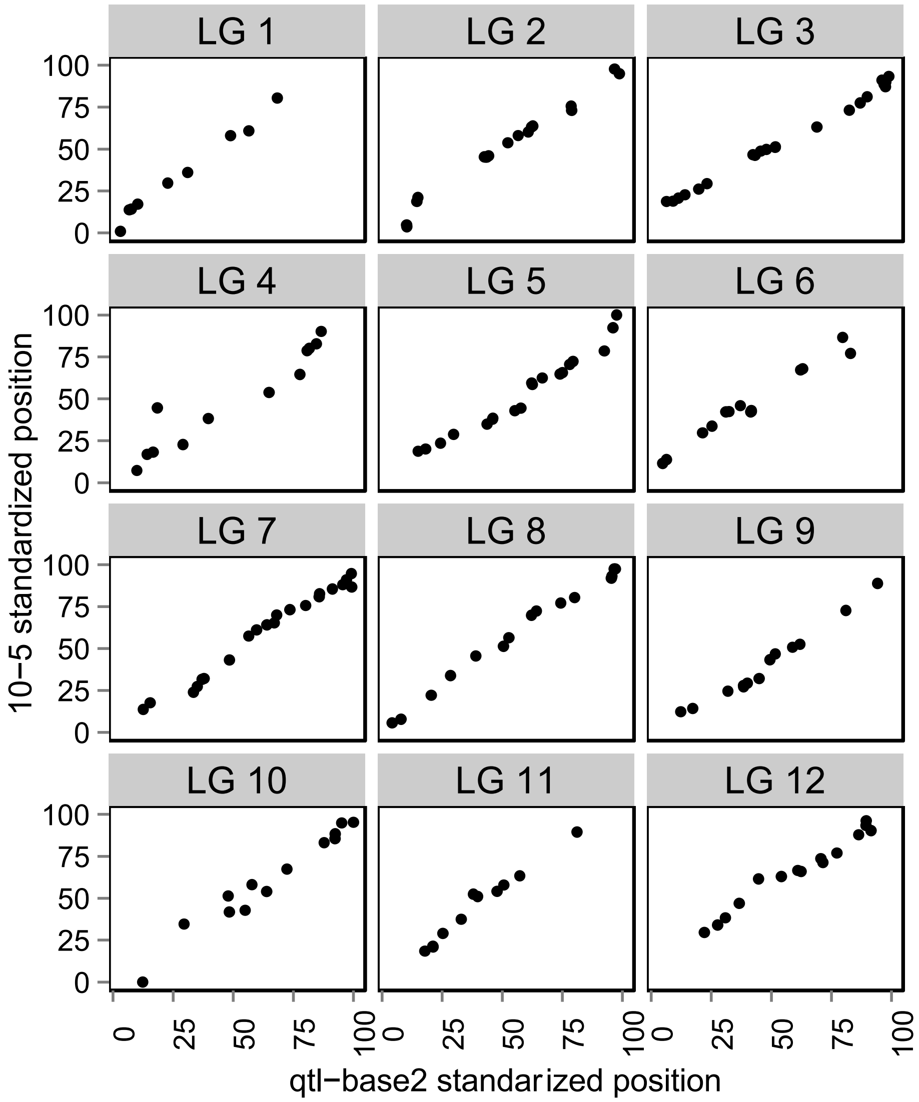

Figure S7 Comparison of order of shared markers between the QTL-BASE2 and reconstructed 10-5 input maps. Linkage group lengths were standardized to 100 units for comparison between maps.

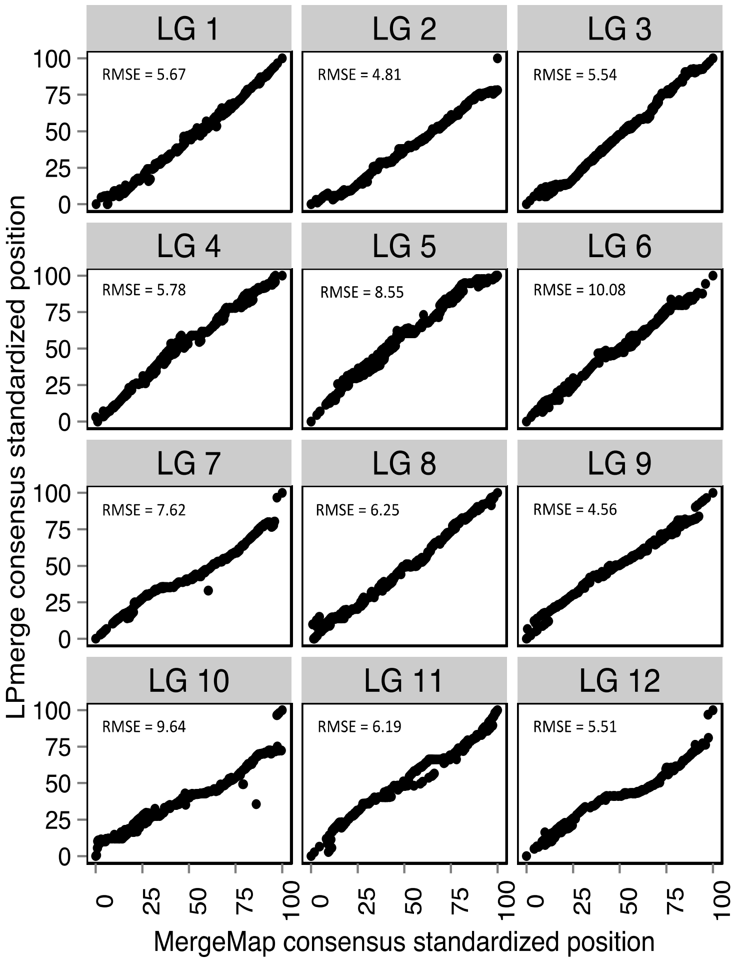

Figure S8 Comparison of order of shared markers between the MergeMap and LPMerge consensus genetic maps for *P. taeda* and *P. elliotti*. Root mean squared error (RMSE) in marker between the consensus maps was estimated for each linkage group.

Figure S9 MapChart of MergeMap consensus genetic map for *P. taeda* and *P. elliottii*
Available for download as a PDF file at www.g3journal.org/lookup/suppl/doi:10.1534/g3.115.019588/-/DC1

Supplementary Tables (S1 – S5)

Table S1 Summary of reconstructed 10-5 input map, locus counts by linkage group and final map goodness-of-fit chi-square

LG	LG Neves *et al.*	*N* loci Neves *et al.*	*N* loci selected	*N* loci start order	*N* loci fixed order	*N* loci mapped	X^2
1	9	223	198	13	6	102	3266
2	11	271	244	16	11	119	2183
3	1	193	175	13	13	113	2422
4	6	230	206	22	10	102	2372
5	8	291	279	11	11	128	3101
6	5	263	253	11	10	121	2461
7	2	260	249	16	9	127	3140
8	7	242	198	15	11	117	1976
9	10	228	214	15	11	115	2457
10	12	240	227	28	19	125	2397
11	4	195	191	13	14	87	2099
12	3	205	204	20	13	119	2873

Table S2 Summary of identity by descent (IBD) proportions among pairs of individuals in the CCLONES pedigree.

IBD proportion	*N* pairs	% of pedigree
0	338676	79.59
0.0625	15545	3.65
0.125	18258	4.29
0.1875	1123	0.26
0.25	43872	10.31
0.3125	1090	0.26
0.375	527	0.12
0.5	6412	1.51

Table S3 Number of markers included and shared among input maps used to construct the *P. taeda* consensus map. The diagonal represents the number of markers selected for inclusion in the consensus map from each input map. The off-diagonal elements represent the number of markers shared between maps.

	10-5	BC1	QTL-BASE1	QTL-BASE2
10-5	1375			
BC1	69	801		
qtl-base1	0	0	406	
qtl-base2	180	497	76	2054

Table S4 Comparisons of the MergeMap and LPmerge *P. taeda* consensus genetic maps.

	MergeMap	LPmerge
Number of markers mapped	3856	3856
Number of unique marker positions	3810	2959
Average marker spacing (cM)	0.599	0.469
Total map length (cM)	2305.42	1802.59
RMSE with input maps averaged across LGs	0.97	1.34

Table S5 Root mean squared error (RMSE) in marker order between the MergeMap or LPmerge consensus maps and the input maps by linkage group (LG). Linkage groups 7 and 12 from the QTL-BASE1 map were not included in the consensus merge due to sharing only two markers shared with the QTL-BASE2 maps, hence RMSE values for these linkage groups are missing (NA).

LG	10-5	BC1	QTL-BASE1	QTL-BASE2	Average RMSE
MergeMap					
1	0	0	0.52	2.54	0.76
2	0	0.57	0.75	1.60	0.73
3	0	0.23	0	2.68	0.73
4	0	0	0	2.34	0.59
5	0	0.59	0.63	1.44	0.66
6	0	1.27	0	3.65	1.23
7	0	0	NA	4.09	1.36
8	0	0.60	0	3.24	0.96
9	0	0.23	0	3.44	0.92
10	0	1.84	1.73	2.31	1.47
11	0	0.98	0.99	0.95	0.73
12	0	0.93	NA	3.47	1.47
LPmerge					
1	0.00	1.76	0	3.38	1.28
2	0.67	0	0.37	2.61	0.92
3	0.13	1.50	0	3.86	1.37
4	0.00	1.53	0	2.61	1.04
5	0.65	0.22	0	1.92	0.70
6	2.50	0.97	0	6.58	2.51
7	0	0	NA	6.61	2.20
8	0.32	1.08	0	2.63	1.01
9	0.32	0.44	0	3.45	1.05
10	0.75	0	0	6.89	1.91
11	0.26	0	0	2.41	0.67
12	0	0.50	NA	3.60	1.37

Files S1-S9

Available for download at www.g3journal.org/lookup/suppl/doi:10.1534/g3.115.019588/-/DC1

File S1 – The QTL-BASE1 linkage map with GIC

File S2 – The QTL-BASE2 linkage map with GIC

File S3 – The BC1 linkage map with GIC

File S4 – The 10-5 linkage map with GIC

File S5 – FASTA formatted expressed sequence tag sequences of mapped markers

File S6 – fastSTRUCTURE results matrix of the ADEPT2 population for three subpopulations

File S7 – MergeMap consensus linkage map for *Pinus taeda* with alignments to the *P. taeda* genome and transcriptome

File S8 – LPmerge consensus linkage map for *Pinus taeda* and *Pinus elliotti*

File S9 – Pairs of expressed sequences containing SNPs in extended LD (R2 >0.1), before and after accounting for structure in ADEPT2 and kinship in CCLONES; table includes MAF and consensus map positions
