# Supplementary material for: A Consensus Genetic Map for Pinus taeda and Pinus elliottii and Extent of Linkage Disequilibrium in Two Genotype-Phenotype Discovery Populations of Pinus taeda
Source: G3 (Bethesda). 2015 Jun 11;5(8):1685–94. doi: 10.1534/g3.115.019588 (PMC4528325; doi:10.1534/g3.115.019588)
Supplement: Supporting Information [file supp_g3.115.019588_FigureS2.pdf]

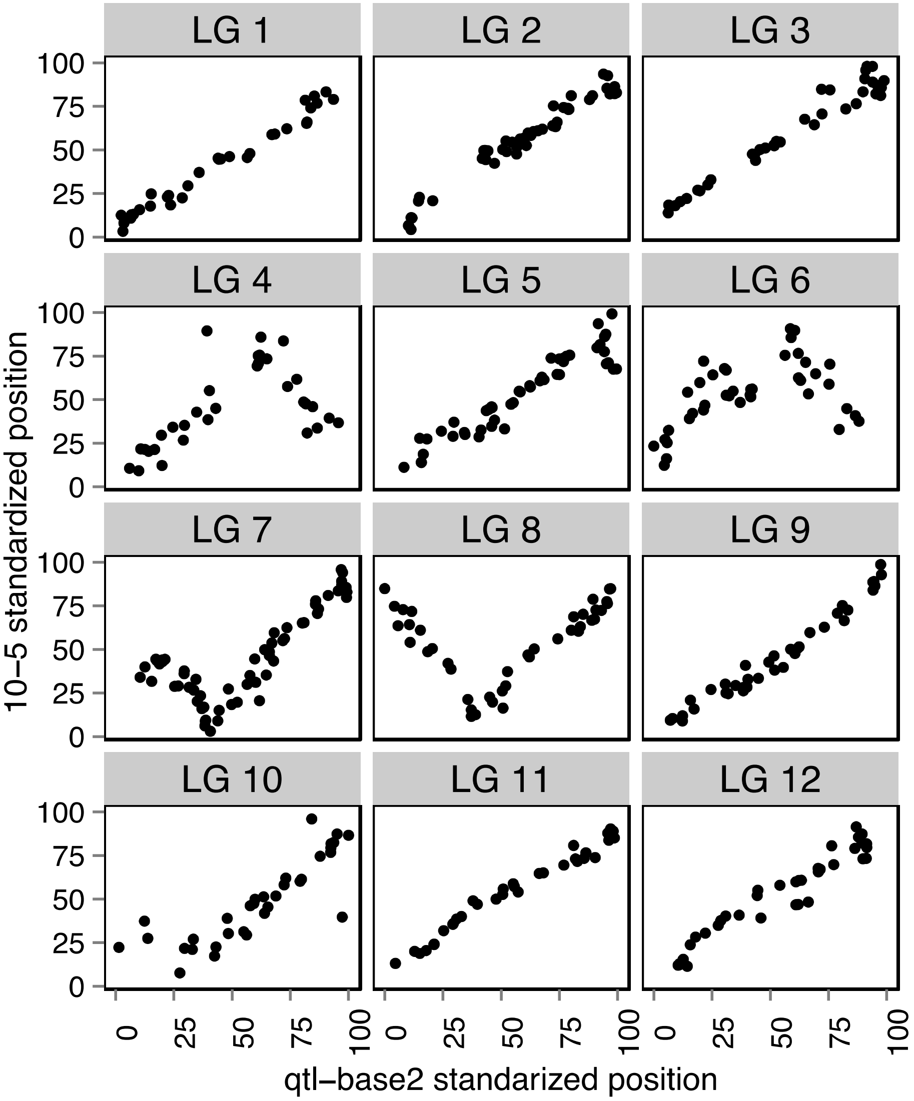

Figure S2 Comparison of order of shared markers between the QTL-BASE2 input map and the original 10-5 from Neves *et al.*, (2014). Linkage group lengths were standardized to 100 units for comparison between maps.
