# Supplementary material for: A Consensus Genetic Map for Pinus taeda and Pinus elliottii and Extent of Linkage Disequilibrium in Two Genotype-Phenotype Discovery Populations of Pinus taeda
Source: G3 (Bethesda). 2015 Jun 11;5(8):1685–94. doi: 10.1534/g3.115.019588 (PMC4528325; doi:10.1534/g3.115.019588)
Supplement: Supporting Information [file supp_g3.115.019588_FigureS5.pdf]

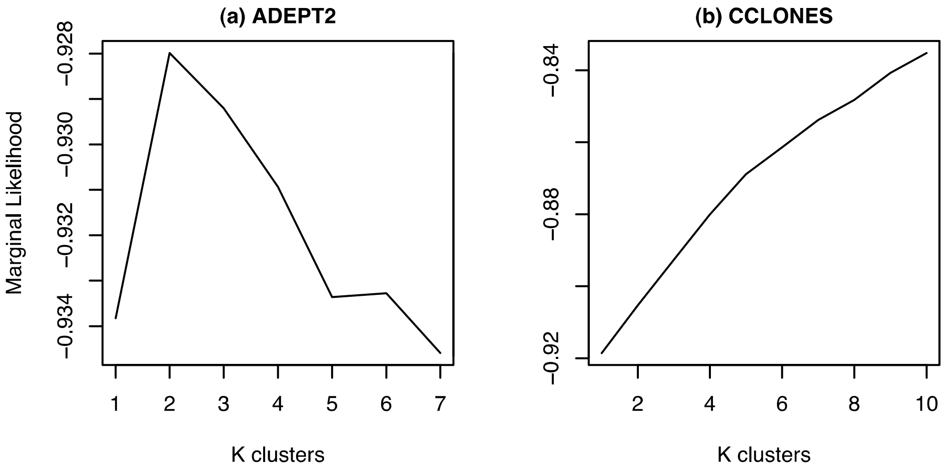

Figure S5 Population structure analysis in ADEPT2 (a), CCLONES (b) with fastSTRUCTURE (Raj et al. 2014). Lines show marginal likelihood at each K
