# Supplementary figures and images for: A Consensus Genetic Map for Pinus taeda and Pinus elliottii and Extent of Linkage Disequilibrium in Two Genotype-Phenotype Discovery Populations of Pinus taeda
Source: G3 (Bethesda). 2015 Jun 11;5(8):1685–94. doi: 10.1534/g3.115.019588 (PMC4528325; doi:10.1534/g3.115.019588)

LG-1

LG-2

LG-3

LG-4

LG-5

LG-6

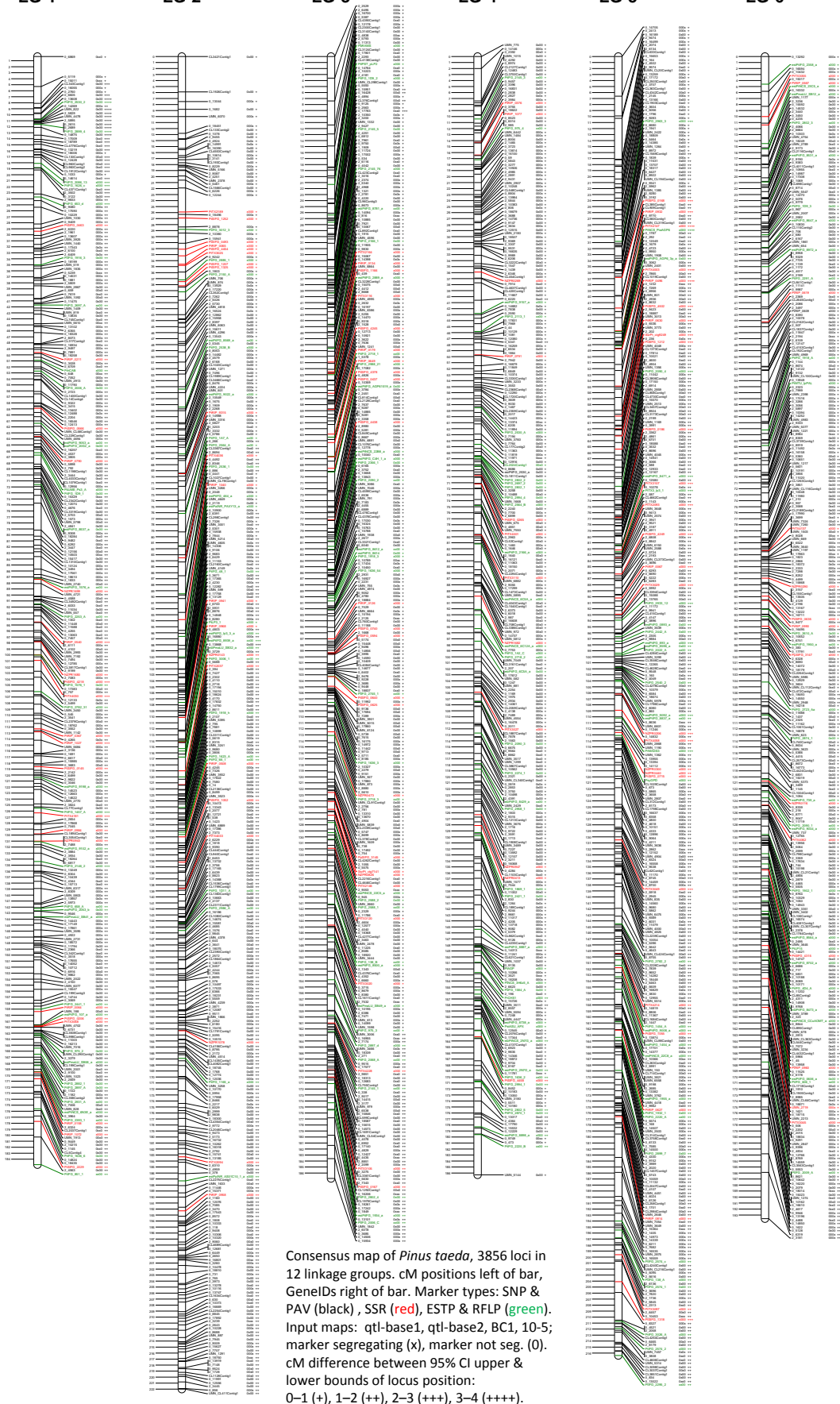

# LG-7

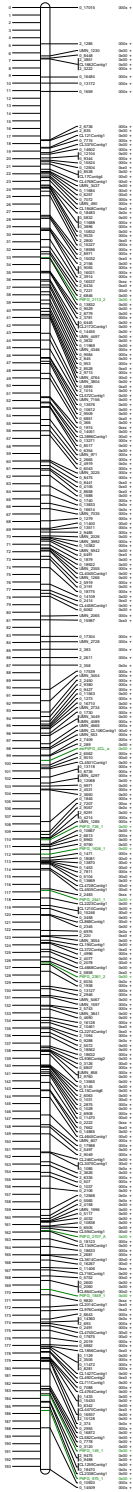

# LG-8

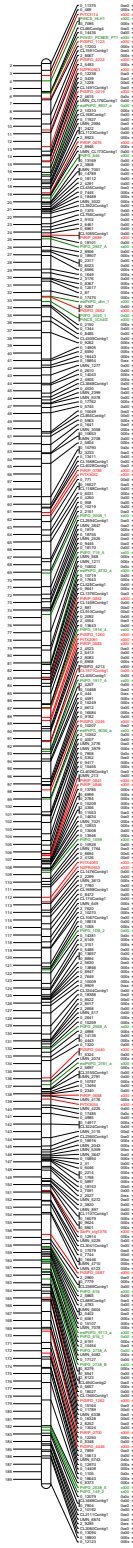

# LG-9

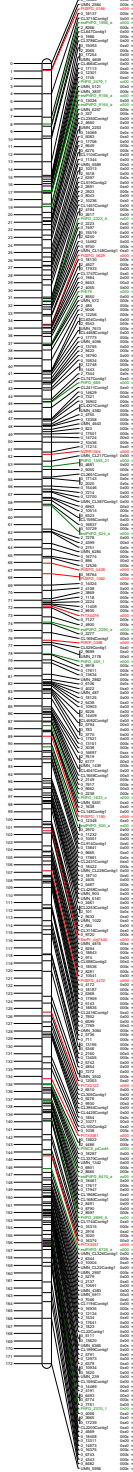

# LG-10

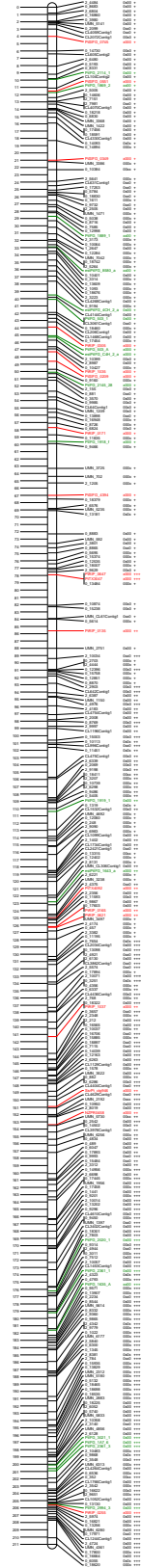

# LG-11

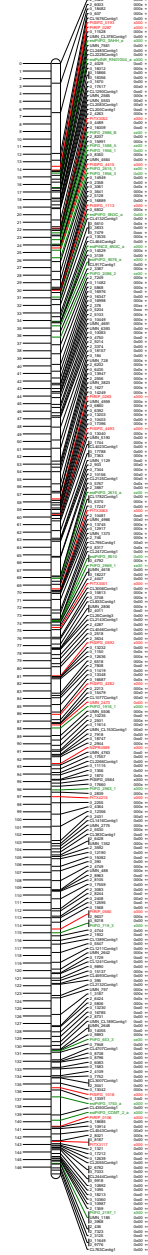

# LG-12

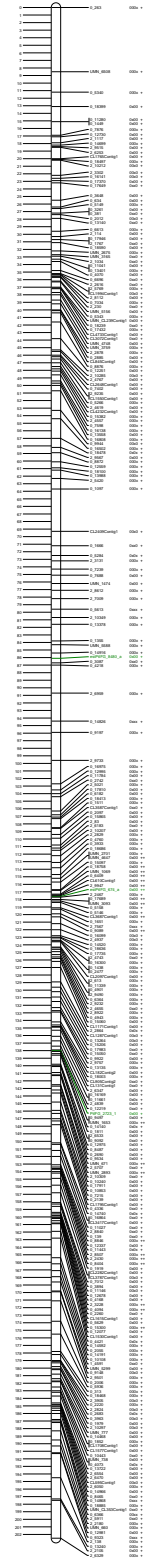

Supplement: Supporting Information [file supp_g3.115.019588_FigureS9.pdf]
