# Supplementary material for: A Consensus Genetic Map for Pinus taeda and Pinus elliottii and Extent of Linkage Disequilibrium in Two Genotype-Phenotype Discovery Populations of Pinus taeda
Source: G3 (Bethesda). 2015 Jun 11;5(8):1685–94. doi: 10.1534/g3.115.019588 (PMC4528325; doi:10.1534/g3.115.019588)
Supplement: Supporting Information [file supp_g3.115.019588_TableS1.pdf]

Supplementary Tables (S1 – S5)

Table S1 Summary of reconstructed 10-5 input map, locus counts by linkage group and final map goodness-of-fit chi-square

LG	LG Neves *et al.*	*N* loci Neves *et al.*	*N* loci selected	*N* loci start order	*N* loci fixed order	*N* loci mapped	X^2
1	9	223	198	13	6	102	3266
2	11	271	244	16	11	119	2183
3	1	193	175	13	13	113	2422
4	6	230	206	22	10	102	2372
5	8	291	279	11	11	128	3101
6	5	263	253	11	10	121	2461
7	2	260	249	16	9	127	3140
8	7	242	198	15	11	117	1976
9	10	228	214	15	11	115	2457
10	12	240	227	28	19	125	2397
11	4	195	191	13	14	87	2099
12	3	205	204	20	13	119	2873
