# Supplementary material for: A Consensus Genetic Map for Pinus taeda and Pinus elliottii and Extent of Linkage Disequilibrium in Two Genotype-Phenotype Discovery Populations of Pinus taeda
Source: G3 (Bethesda). 2015 Jun 11;5(8):1685–94. doi: 10.1534/g3.115.019588 (PMC4528325; doi:10.1534/g3.115.019588)
Supplement: Supporting Information [file supp_g3.115.019588_TableS2.pdf]

Table S2 Summary of identity by descent (IBD) proportions among pairs of individuals in the CCLONES pedigree.

IBD proportion	*N* pairs	% of pedigree
0	338676	79.59
0.0625	15545	3.65
0.125	18258	4.29
0.1875	1123	0.26
0.25	43872	10.31
0.3125	1090	0.26
0.375	527	0.12
0.5	6412	1.51
