# Supplementary material for: A Consensus Genetic Map for Pinus taeda and Pinus elliottii and Extent of Linkage Disequilibrium in Two Genotype-Phenotype Discovery Populations of Pinus taeda
Source: G3 (Bethesda). 2015 Jun 11;5(8):1685–94. doi: 10.1534/g3.115.019588 (PMC4528325; doi:10.1534/g3.115.019588)
Supplement: Supporting Information [file supp_g3.115.019588_TableS3.pdf]

Table S3 Number of markers included and shared among input maps used to construct the *P. taeda* consensus map. The diagonal represents the number of markers selected for inclusion in the consensus map from each input map. The off-diagonal elements represent the number of markers shared between maps.

	10-5	BC1	QTL-BASE1	QTL-BASE2
10-5	1375			
BC1	69	801		
qtl-base1	0	0	406	
qtl-base2	180	497	76	2054
