# Supplementary material for: A Consensus Genetic Map for Pinus taeda and Pinus elliottii and Extent of Linkage Disequilibrium in Two Genotype-Phenotype Discovery Populations of Pinus taeda
Source: G3 (Bethesda). 2015 Jun 11;5(8):1685–94. doi: 10.1534/g3.115.019588 (PMC4528325; doi:10.1534/g3.115.019588)
Supplement: Supporting Information [file supp_g3.115.019588_TableS4.pdf]

Table S4 Comparisons of the MergeMap and LPmerge *P. taeda* consensus genetic maps.

	MergeMap	LPmerge
Number of markers mapped	3856	3856
Number of unique marker positions	3810	2959
Average marker spacing (cM)	0.599	0.469
Total map length (cM)	2305.42	1802.59
RMSE with input maps averaged across LGs	0.97	1.34
