# Supplementary material for: A Consensus Genetic Map for Pinus taeda and Pinus elliottii and Extent of Linkage Disequilibrium in Two Genotype-Phenotype Discovery Populations of Pinus taeda
Source: G3 (Bethesda). 2015 Jun 11;5(8):1685–94. doi: 10.1534/g3.115.019588 (PMC4528325; doi:10.1534/g3.115.019588)
Supplement: Supporting Information [file supp_g3.115.019588_TableS5.pdf]

Table S5 Root mean squared error (RMSE) in marker order between the MergeMap or LPmerge consensus maps and the input maps by linkage group (LG). Linkage groups 7 and 12 from the QTL-BASE1 map were not included in the consensus merge due to sharing only two markers shared with the QTL-BASE2 maps, hence RMSE values for these linkage groups are missing (NA).

LG	10-5	BC1	QTL-BASE1	QTL-BASE2	Average RMSE
MergeMap					
1	0	0	0.52	2.54	0.76
2	0	0.57	0.75	1.60	0.73
3	0	0.23	0	2.68	0.73
4	0	0	0	2.34	0.59
5	0	0.59	0.63	1.44	0.66
6	0	1.27	0	3.65	1.23
7	0	0	NA	4.09	1.36
8	0	0.60	0	3.24	0.96
9	0	0.23	0	3.44	0.92
10	0	1.84	1.73	2.31	1.47
11	0	0.98	0.99	0.95	0.73
12	0	0.93	NA	3.47	1.47
LPmerge					
1	0.00	1.76	0	3.38	1.28
2	0.67	0	0.37	2.61	0.92
3	0.13	1.50	0	3.86	1.37
4	0.00	1.53	0	2.61	1.04
5	0.65	0.22	0	1.92	0.70
6	2.50	0.97	0	6.58	2.51
7	0	0	NA	6.61	2.20
8	0.32	1.08	0	2.63	1.01
9	0.32	0.44	0	3.45	1.05
10	0.75	0	0	6.89	1.91
11	0.26	0	0	2.41	0.67
12	0	0.50	NA	3.60	1.37
